# Artificial intelligence-based structural modeling of PSTOL1 receptor kinases in rice and sorghum

**DOI:** 10.3389/fpls.2026.1850847

**Published:** 2026-09-07

**Authors:** Marcos José Andrade Viana, Natália Florêncio Martins, Leandro Carrijo Cintra, Beatriz de Almeida Barros, Raquel Cardoso de Melo Minardi, Jurandir Vieira Magalhaes

**Affiliations:** 1 Graduate Program in Bioinformatics, Federal University of Minas Gerais (UFMG), Belo Horizonte, MG, Brazil; 2 Embrapa Tropical Agroindustry, Fortaleza, CE, Brazil; 3 Embrapa Digital Agriculture, Campinas, SP, Brazil; 4 Embrapa Maize and Sorghum, Sete Lagoas, MG, Brazil

**Keywords:** kinase activation, oligomerization, phosphorus efficiency, plant signaling, structural modeling

## Abstract

Originally identified in rice (*Oryza sativa* OsPSTOL1), PHOSPHORUS-STARVATION TOLERANCE 1 (PSTOL1) proteins are essential determinants of phosphorus efficiency in several plant species, including *Sorghum bicolor* (SbPSTOL1). In contrast to OsPSTOL1, SbPSTOL1 homologs exhibit a transmembrane receptor-like kinase (RLK) architecture with polymorphic extracellular domains. We employed an AI-driven computational pipeline to investigate the structure and conformational dynamics of SbPSTOL1 proteins. Monomeric predictions using multiple deep-learning platforms yielded structures that were inconsistent with the SbPSTOL1 RLK topology, suggesting inherent monomeric instability or autoinhibition. In a validation step, we also modeled the phylogenetically related ZmWAKL RLK, which forms a heterodimer with its likely co-receptor, ZmWIK, and reproduced their interaction *in silico*. Oligomeric modelling resulted in structurally plausible models for SbPSTOL1 proteins that overcame the structural monomeric instability. Molecular dynamics simulations in a membrane environment also supported the preferential stability of oligomeric SbPSTOL1 states. Regional analysis suggested that, while the transmembrane and kinase domains are more rigid, the extracellular regions exhibit higher flexibility, consistent with their roles in environmental perception. Notably, oligomerization was associated with a conformational transition in the catalytic site, likely enabling the formation of the canonical αC-Glu ↔ β3-Lys salt bridge, a hallmark of kinase activation. Altogether, these results support a model in which oligomerization is a probable structural prerequisite for the catalytic competence of SbPSTOL1 but not necessarily of OsPSTOL1. These AI-based findings represent testable hypotheses for future experimental validation and present the first *in silico* structural characterization of SbPSTOL1 proteins, offering insights for elucidating complex RLK-mediated plant signaling mechanisms.

## 1 Introduction

Global food security faces an unprecedented challenge due to the intensification of climate change, driven by a projected population increase to 9.7 billion by 2050. The Food and Agriculture Organization (FAO) estimates that food production must increase by 70% to meet this continuously growing demand, a goal hindered by increasing exposure of crops to abiotic stresses (Challinor et al., 2010; Fedoroff et al., 2010; Zhu, 2016). In current climate change scenarios, plant exposure to abiotic factors on acidic soils (pH < 5.5), which cover up to 50% of the world’s arable land (von Uexküll and Mutert, 1995), represents a major bottleneck for agricultural productivity. On acidic soils, which are typically located in tropical and subtropical regions, crop production is severely limited by a complex of co-occurring abiotic stress factors, including aluminum toxicity and, crucially, low phosphorus (P) availability (Lynch, 2019; Kochian et al., 2015; Foy, 1984). Phosphorus, an essential macronutrient for energy metabolism and photosynthesis (Raghothama, 1999), becomes largely unavailable on acidic soils because it binds strongly to iron and aluminum oxides in the soil clay fraction (Hinsinger, 2001; Sanchez, 1976). Consequently, developing cultivars with enhanced P acquisition efficiency (PAE) is imperative for sustainable agriculture (Lynch, 2019) and, in consequence, is a major determinant of food security worldwide.

Sorghum (*Sorghum bicolor* L. Moench), a C4 crop that is resilient to different environmental challenges, serves as an excellent model for dissecting crop abiotic stress tolerance (Tari et al., 2012). Phosphorus Use Efficiency (PUE) in sorghum is typically partitioned into Phosphorus Acquisition Efficiency (PAE) and internal Phosphorus Utilization Efficiency (PUTIL). Recent studies have demonstrated that PAE is the primary driver of sorghum grain yield under low P availability in the soil (Bernardino et al., 2019).

A milestone in understanding PAE was the identification of the *PHOSPHORUS-STARVATION TOLERANCE 1* (*OsPSTOL1*) gene in rice (*Oryza sativa*), which was predicted to be a cytosolic kinase and shown to enhance root growth and P acquisition (Gamuyao et al., 2012). In sorghum, association mapping revealed that *SbPSTOL1* homologs are also associated with improved root morphology and grain yield under low P availability in the soil (Hufnagel et al., 2014; Bernardino et al., 2019; Bernardino et al., 2021). Subsequently, *PSTOL1* genes have been shown to enhance root morphology in other species. In maize (*Zea mays*), *PSTOL1* homologs were identified and associated with QTLs for root surface area and biomass accumulation, performing functions similar to *OsPSTOL1* (Azevedo et al., 2015). The promoter of the wheat (*Triticum aestivum*) PSTOL1 gene, *TaPSTOL*, was induced in root tips and root hairs upon P starvation and genetic perturbation of *TaPSTOL* influenced different traits, although this was not followed by an increase in PUE (Milner et al., 2023). Functional conservation of the PSTOL1 family across species was further supported by heterologous expression of *SbPSTOL1* in *Arabidopsis thaliana* and tobacco (*Nicotiana tabacum*). These studies demonstrated that PSTOL1 homologs significantly modulate root system architecture, leading to increased surface area and enhanced P acquisition efficiency (Mukkawar et al., 2024; Maciel et al., in preparation). However, unlike the cytosolic nature of the receptor-like cytoplasmic kinase (RLCK) OsPSTOL1 in rice, *SbPSTOL1* proteins show strikingly different topologies and are, on this basis, true RLKs. Protein domain analysis followed by cellular localization studies indicate that *SbPSTOL1* proteins are transmembrane Receptor-Like Kinases (RLKs) and are polymorphic for the presence of functional cell wall association domains (Hufnagel et al., 2014; Maciel et al., in preparation).

Plant RLKs, with their typical modular tripartite architecture (Shiu and Bleecker, 2001), are fundamental signal transducers in many biotic and abiotic stress pathways. The RLK topology comprises an extracellular ligand-binding domain (ECD) responsible for signal perception, a single single-pass transmembrane (TM) α-helix and a conserved cytoplasmic kinase domain (KD), usually a serine/threonine kinase that propagates signal via phosphorylation. Although this basic organization is ubiquitous in plant RLKs (Dievart et al., 2020), these proteins are markedly polymorphic in their architecture. Variations in the ECD, such as in the Leucine-Rich Repeat (LRR) or Lysin Motif (LysM) domains, enable detection of a broad spectrum of stimuli, ranging from endogenous hormones to pathogen-associated molecular patterns (PAMPs) (Shiu and Bleecker, 2001). Canonically, receptor activation is initiated by ligand binding to the ECD, which stabilizes the formation of homo- or hetero-oligomeric complexes (Hohmann et al., 2017). This enables reciprocal trans-phosphorylation, where the kinase domain of one monomer catalyzes the phosphorylation of specific residues on its partner (Hohmann et al., 2017). These events trigger further autophosphorylation across the receptor complex, alleviating autoinhibitory constraints and establishing a catalytically competent state for signal propagation to downstream substrates (Hohmann et al., 2017). Notably, however, PSTOL1 kinases, including OsPSTOL1, the SbPSTOL1 proteins and the phylogenetically related ZmWAKL, belong to the non-RD class of kinases (Dardick and Ronald, 2006), which lack the conserved arginine immediately preceding the catalytic aspartate and are frequently associated with distinct, non-canonical regulatory mechanisms; their activation is therefore not expected to strictly follow the canonical RD-kinase paradigm (Dardick et al., 2012).

The catalytic core of plant RLKs is organized in a conserved kinase domain composed of two distinct segments, a smaller N-terminal lobe (N-lobe), primarily consisting of β-strands, and a larger, mainly helical C-terminal lobe (C-lobe). These two lobes are connected by a flexible hinge region, forming a deep cleft that houses the catalytic site where ATP and substrate binding occur (Huse and Kuriyan, 2002). Precise communication between these lobes is essential for enzymatic function, as the relative orientation of specific secondary structure elements within the N-lobe, namely the αC-helix and the β3-strand, dictates whether the kinase is in an active or inactive conformation (Endicott et al., 2012).

The transition to a catalytically competent state of the kinase domain depends on a molecular switch shared by eukaryotic kinases (Taylor and Kornev, 2011; Endicott et al., 2012). Central to this mechanism is the conformational movement of the αC-helix, which transitions from an external position (αC-out) to an internal position (αC-in). At cytosolic pH, this shift enables the formation of a critical stabilizing salt bridge, an electrostatic interaction between the conserved positively charged (–NH_3_^+^) Lysine in the β3-strand (β3-Lys) and the negatively charged (–COO^−^) Glutamate residue in the αC-helix (αC-Glu). The electrostatic attraction between these residues (αC-Glu ↔ β3-Lys, where the symbol ↔ denotes a functional salt bridge interaction) stabilizes the ATP-binding pocket in the N-lobe of the protein, correctly orienting phosphate for catalytic transfer (Taylor and Kornev, 2011). Without the precise positioning of the αC-helix, the kinase domain remains incapable of processing its ATP substrate, even if it is present.

In the inactive state, however, this mechanism is hindered by autoinhibitory constraints that prevent premature activation. Such restrictions may involve the activation loop, which projects over the catalytic site obstructing substrate entry and maintaining the αC-helix in the inactive conformation (Endicott et al., 2012). Phosphorylation of specific residues within the activation loop alleviates this repression, promoting a conformational rearrangement that stabilizes the αC-helix in the active position, granting substrate access to the catalytic cleft (Liu et al., 2024). Nevertheless, the specific structural mechanism governing the transition between inhibited and active states of *PSTOL1* proteins remains unknown.

Structural modeling of Receptor-Like Kinases (RLKs) presents inherent challenges due to the structural complexity of membrane-bound proteins (Hohmann et al., 2017; Hegedűs et al., 2022; Lomize et al., 2022). While it is unquestionable that AI-based approaches were a significant breakthrough in protein modelling (Jumper et al., 2021), a critical obstacle lies in the interpretation of automated confidence scores. Metrics such as the Predicted Local Distance Difference Test (pLDDT) estimates local model precision by comparing predicted interatomic distances against a statistical distribution of experimentally solved structures in the Protein Data Bank (PDB) (Jumper et al., 2021), providing valuable support of local geometry. Nevertheless, these metrics are often insufficient to validate the biological viability of complex RLK architectures (Hegedűs et al., 2022). Biologically, the functional integrity of RLKs requires rigorous spatial segregation between the extracellular (ECD) and kinase (KD) domains, which is maintained by the lipid bilayer. Therefore, the validation of predicted models must transcend purely statistical metrics, integrating knowledge of cellular topology and the conservation of functional motifs (Jumper et al., 2021). Without this contextualization, models exhibiting high confidence scores may nonetheless display “topologically collapsed” conformations that are physically incompatible with the membrane environment and inconsistent with known RLK architectures.

Given this complexity, the maize (*Zea mays*) ZmWAKL–ZmWIK heterodimer was used to validate our prediction pipeline, as its physical interaction and reciprocal trans-phosphorylation have been experimentally demonstrated (Zhong et al., 2024). This choice was based on the phylogenetic proximity of ZmWAKL to SbPSTOL1 (Maciel et al., in preparation), as well as the essential role of the ZmWAKL/ZmWIK heterodimer in development and stress responses in cereals (Zhong et al., 2024). Using Alphafold3 (Abramson et al., 2024), we have been able to accurately reproduce *in silico* the formation of the ZmWAKL/ZmWIK complex, while maintaining spatial segregation between the ECD and KD.

The objective of this study was to apply an AI-driven framework to model SbPSTOL1 structure to reveal patterns potentially critical for catalytic competence, such as structural compatibility with the formation of the canonical αC-Glu ↔ β3-Lys salt bridge. To overcome the limitations of static predictions, the resulting models were further evaluated using molecular dynamics simulations. Together, these analyses provide structural insights into the SbPSTOL1 function.

## 2 Materials and methods

We developed an integrative, AI-driven computational pipeline tailored for large receptor-like kinases (RLKs) to systematically characterize the structural properties of SbPSTOL1 proteins. This framework combines complementary strategies that are rarely applied in concert to characterize plant RLKs.

First (1), we performed detailed sequence and functional annotations to map conserved domains and regulatory motifs; (2) Next, we implemented a multimodal structural modeling strategy, integrating predictions of multiple state-of-the-art deep-learning platforms to predict both monomeric and oligomeric conformations; (3) Crucially, we advanced beyond conventional static modeling by incorporating a comprehensive stability assessment, including membrane embedding, quantitative interface analysis and region-resolved molecular dynamics simulations. This integrative approach enabled us to address the topological and conformational challenges inherent to membrane-bound, large receptor-like kinases (RLKs), allowing the discrimination between structurally unstable and biologically plausible models. By coupling AI-based structural predictions with dynamic and interface-level validation, our pipeline provides a high-resolution, time-resolved framework for evaluating receptor activation mechanisms. More broadly, it establishes a transferable computational strategy for dissecting complex signaling architectures in plant receptor kinases. An overview of the complete computational pipeline is provided in **Supplementary Figure 1**.

### 2.1 Sequence analysis and functional domains

The amino acid sequences of three *SbPSTOL1* homologs, Sb03g006765, Sb03g031690 and Sb07g002840 (Hufnagel et al., 2014) were retrieved from v1.4 of the sorghum genome available in the Phytozome database (https://phytozome-next.jgi.doe.gov/). *The Sb03g006765 sequence starts with the first ATG located 72 bp upstream of the first codon (TCA) in v1.4 (Hufnagel et al., 2014).* In the most recent genome version, v5.1, the corresponding gene identifiers are *Sobic.003G080700*, *Sobic.003G263400* and *Sobic.007G032900*, respectively (Maciel et al., in preparation). The amino acid sequences of ZmWAKL, ZmWIK (Zhong et al., 2024) and OsPSTOL1 (Gamuyao et al., 2012) were retrieved from the National Center for Biotechnology Information (NCBI) and their identifiers are OQ421108, OQ421112 and BAK26566, respectively.

*In silico* functional characterization and domain annotation of RLK proteins were performed using InterProScan (Blum et al., 2020). This platform integrates multiple databases, including Pfam, SMART, SUPERFAMILY, CDD and PROSITE as well as tools for predicting transmembrane domains and signal peptides, PHOBIUS and TMHMM. Predictions for transmembrane helices and subcellular localization were also confirmed using InterProScan.

The conserved catalytic sites within the kinase domain, including the ATP binding site (InterPro: IPR017441) and the serine/threonine active site (InterPro: IPR008271), were identified. The activation loop, a key regulatory element of protein kinases, was manually identified in each sequence by locating the canonical DFG (start) and APE (end) motifs (Hanks and Hunter, 1995; Gógl et al., 2019).

The amino acid sequences of PSTOL1 proteins were aligned using Clustal Omega (Sievers et al., 2011). The resulting alignment file was processed with the ESPript 3.0 (Robert and Gouet, 2014) web server (https://espript.ibcp.fr/ESPript/ESPript/) to generate a graphical representation of conserved residues and the sequence of predicted structural motifs. Structural information was derived from AI-based three-dimensional models as described below, which were used to identify α-helices and β-strands onto the sequence alignment.

### 2.2 Structural modeling and validation strategy

Deep learning-based structural modeling was conducted on the High-Performance Computing (HPC) infrastructure of the Multi-User Bioinformatics Laboratory (LMB) at Embrapa Digital Agriculture (Campinas, SP, Brazil). AlphaFold3 predictions were undertaken on the web server, https://alphafoldserver.com/.

#### 2.2.1 Monomeric modeling

The algorithms implemented in AlphaFold2 (versions 2.1, 2.2 and 2.3) and AlphaFold3 were executed using default parameters. For AlphaFold2, the “full_dbs” option was utilized, encompassing the databases, UniRef90, MGnify, BFD, Uniclust30 and PDB70. Predictions with RoseTTAFold (v1 and v2) and RoseTTAFold-All-Atom were also conducted using default settings, employing the recommended sequence search pipelines against the UniRef30 and BFD databases.

#### 2.2.2 Oligomer modeling

Potential heterodimers and one heterotrimer of SbPSTOL1 proteins were modelled. Oligomer predictions were generated using AlphaFold-multimer, (Evans et al., 2021) RoseTTAFold2, RoseTTAFold-All-Atom and AlphaFold3. AlphaFold-multimer was executed using the “full_dbs” option, while all other algorithms were run with their default parameters.

The viability of the resulting models was assessed based on topological criteria. A model was considered viable only if it exhibited spatial segregation of extracellular (ECD), transmembrane and intracellular domains, without spurious physical contacts between domains. For example, a direct contact between the ECD and the kinase domain, which are separated by the plasma membrane, was considered not viable and the respective predictions were deemed inconsistent.

In light of the known structural basis of RLK activation, a validation step was established using the maize ZmWAKL–ZmWIK complex (Zhong et al., 2024). The use of this known interacting pair allowed for an evaluation of the algorithm’s ability, particularly AlphaFold3, to accurately predict the spatial arrangement of membrane-bound architectures. Modeling of the sorghum homologs proceeded only after confirming that the computational pipeline correctly reproduced a biologically viable topology for the ZmWAKL–ZmWIK complex.

### 2.3 Molecular dynamics simulations and interface characterization

We studied how PSTOL1 proteins move across time via Molecular Dynamics Simulations, which was done *in silico* by embedding the RLK proteins into a lipid bilayer followed by detailed simulations of atomic motions over time.

#### 2.3.1 Membrane insertion

Both monomeric and oligomeric models were computationally embedded into a hydrophobic, 1-palmitoyl-2-oleoyl-phosphatidylcholine (POPC) lipid bilayer using the CHARMM-GUI Membrane Builder (Jo et al., 2008). To ensure biological accuracy, the membrane boundaries and the tilt of the protein within the bilayer were predicted using the PPM 2.0 Server (Lomize et al., 2011) integrated into the CHARMM-GUI environment. The predicted membrane coordinates were cross-validated with the domain annotations previously obtained with InterProScan, which defined the spatial arrangement of the extracellular, transmembrane and cytoplasmic regions. This integrated approach allowed the transmembrane helices to attain their most likely biological conformation, providing the necessary environment for subsequent structural analysis.

#### 2.3.2 Interface analysis

The region where two molecules are predicted to interact, designated intermolecular interfaces, and the physicochemical forces stabilizing interfaces were quantified using the COCaDA (Contact Detection Algorithm) software (Lemos et al., 2025). The software identifies non-covalent interactions based on specific Euclidean distance (Da) and geometric cutoffs established in the literature for protein-protein interfaces:

- Hydrogen bonds and salt bridges were identified using a threshold of 0 ≤ Da ≤ 4 Å, with the latter requiring the additional presence of hydrogen bonding between equally charged atoms.
- Hydrophobic interactions were defined within the 2.0 ≤ Da ≤ 4.5 Å interval among hydrophobic atom groups.
- Electrostatic forces: Attractive interactions were monitored within 3.9 ≤ Da ≤ 6.0 Å, while repulsive forces were defined within 2.0 ≤ Da ≤ 6.0 Å to evaluate the distribution of charges across the interface.
- Aromatic stacking: identified by the proximity (2.0 ≤ Da ≤ 5.0 Å) of the centers of aromatic rings (i.e. centroids). These were classified into two geometries: face-to-face (parallel rings) or edge-to-face, where the edge of one ring interacts with the face of another.

Furthermore, we measured the distance between conserved residues of the canonical salt bridge (αC-Glu ↔ β3-Lys) across all models to evaluate the structural indicators of the kinase domain activation state. All structures underwent technical curation via visual inspection to ensure absence of atomic overlaps or topological distortions, with images generated using PyMOL (Schrödinger, LLC 2021). When likely artifacts were detected, the model was discarded.

#### 2.3.3 Molecular dynamics

To evaluate the conformational dynamics and structural integrity of the SbPSTOL1 proteins, molecular dynamics simulations were performed. This computational approach enables time-resolved characterization of individual atomic-level motions under predefined conditions over time, which simulates those under physiological conditions. The workflow was divided into four main stages, with the CHARMM-GUI Membrane Builder (Jo et al., 2008), which provided the necessary input files and parameters for the NAMD3 (Phillips et al., 2020) simulation engine:

1. System Preparation (CHARMM-GUI): the protein models were embedded into a POPC lipid bilayer, solvated with water molecules and neutralized with 0.15 M KCl ions (Jo et al., 2008). This stage established the initial configuration for the simulations.
2. Energy Minimization (NAMD3): an initial optimization was performed to eliminate steric clashes (atomic overlaps) and ensure the system reached a stable starting energy level (Phillips et al., 2020).
3. Equilibration and Heating (NAMD3): the system was heated to 303.15 K. Harmonic restraints used to maintain atoms near their reference positions were gradually released to allow the protein and lipids to adapt to the membrane environment.
4. Production (NAMD3): this is the final data collection stage. The system was simulated in the NPT ensemble (under constant pressure and temperature) using the CHARMM36m (Huang et al., 2016) force field to mimic the cellular environment (Phillips et al., 2020).

Simulation lengths in nanoseconds were determined by the structural convergence of the Root Mean Square Deviation (RMSD), calculated as the average atomic displacement relative to the initial equilibrated frame.

#### 2.3.4 Regionalized stability analysis

To prevent high-amplitude natural motions of the flexible extracellular domains from masking subtle but critical structural transitions within the transmembrane and kinase regions, a regionalized analysis was implemented using customized TCL scripts within the Visual Molecular Dynamics (VMD) environment (Humphrey et al., 1996).

Unlike a standard global RMSD (Root Mean Square Deviation), which measures the overall structural deviation of the entire complex, the systems were partitioned into three regions, extracellular, transmembrane and intracellular. For each region, an independent least squares-based alignment was performed using the initial equilibrated structure as a reference. This procedure effectively removed global rotational and translational movements for each specific domain, allowing for a precise calculation of internal RMSD and RMSF (Root Mean Square Fluctuation) (Martínez, 2015). RMSF measures how much each atom or residue fluctuates around its average position during a trajectory.

This “decoupling” approach, executed via VMD, was applied to isolate the intrinsic stability of the receptor interfaces and catalytic motifs. By evaluating residue-level flexibility with RMSF, we sought more accurate biological insights into complex dynamics, specifically identifying regions that are likely stable versus highly mobile within the signaling complex (Martínez, 2015).

## 3 Results

### 3.1 Analysis of *SbPSTOL1* domains

**Figure 1:**
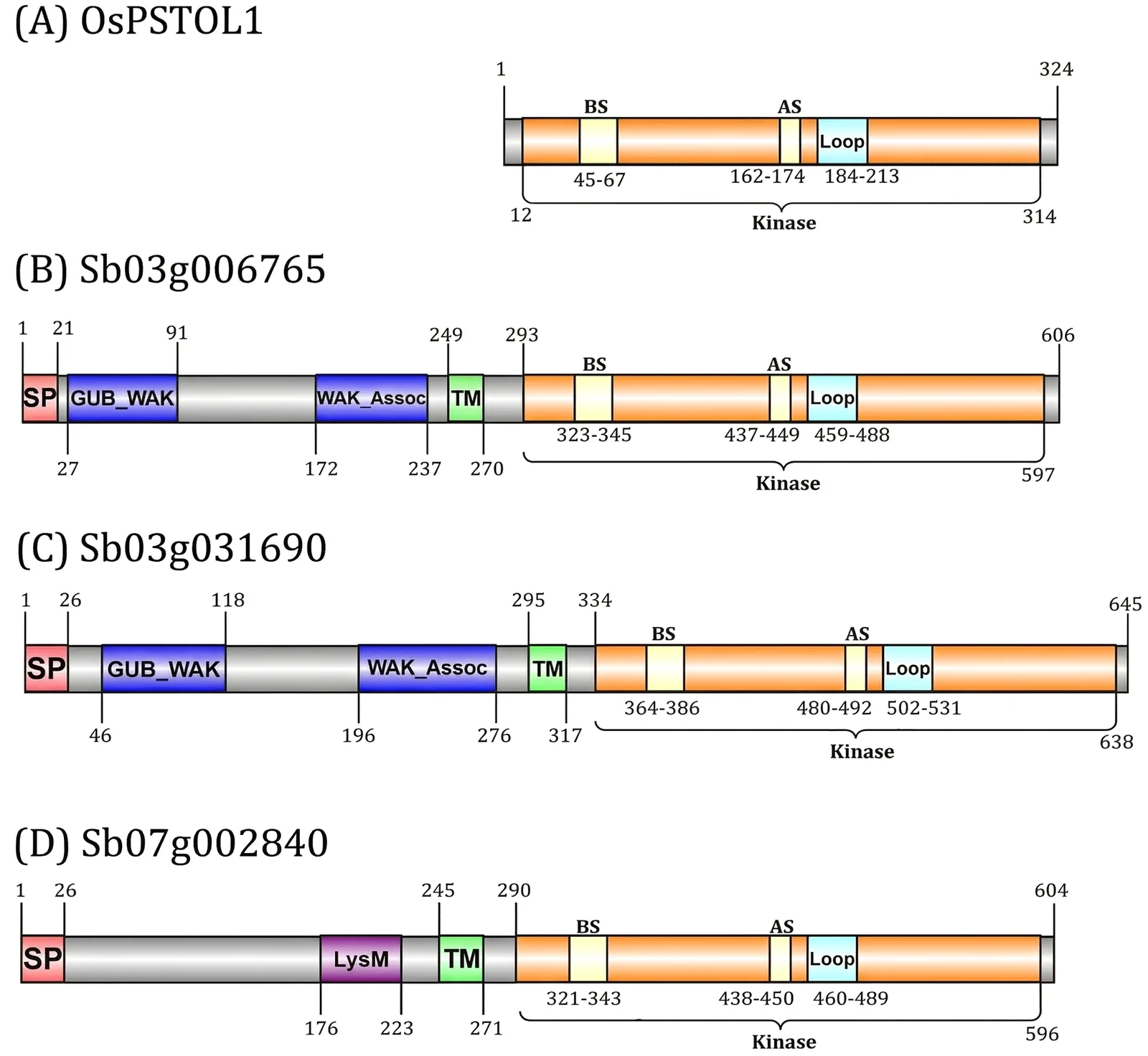
Domain architecture of PSTOL1 homologs in rice and sorghum. The schematic representation of the domain architecture (**Figure 1**) was drawn using IBS (Liu et al., 2015) **(A)** OsPSTOL1, **(B)** Sb03g006765, **(C)** Sb03g031690 and **(D)** Sb07g002840. The sorghum proteins contain an N-terminal signal peptide (SP, red) and a single transmembrane helix (TM, green) which are absent in OsPSTOL1. The conserved kinase domain (orange) features the active site (AS) and the ATP_binding site (BS) (yellow) and the activation loop (cyan). Extracellularly, Sb03g006765 and Sb03g031690 possess the WAK-like domains, Galacturonan-binding Wall-Associated Kinase binding (GUB_WAK) and the Wall-Associated Kinase Associated (WAK_Assoc), while Sb07g002840 features a LysM domain (receptor-like kinase family LYRK3, RLK10).

The three sorghum *SbPSTOL1* proteins, Sb03g006765, Sb03g031690 and Sb07g002840 exhibit a tripartite architecture comprising an N-terminal signal peptide, a single-pass transmembrane (TM) helix and a conserved intracellular kinase domain, typical of RLK proteins (**Figure 1**). In contrast, the rice protein OsPSTOL1 consists solely of a cytosolic catalytic domain, lacking any membrane-anchoring motifs.

Structural divergence among the sorghum homologs lies primarily in the extracellular domains. Sb03g006765 and Sb03g031690 share the wall-associated kinase (WAK) domains, GUB_WAK and WAK_Assoc, which are known to anchor the receptor kinases to the cell wall (Kohorn and Kohorn, 2012). Conversely, only in Sb07g002840 there is an extracellular LysM domain, specialized in recognizing glycans and microbe-associated molecular patterns (Antolín-Llovera et al., 2012).

Detailed analysis of the intracellular kinase domain revealed that both the total size of the KDs, which varies only slightly in size, between 303 and 307 amino acids, and the internal architecture of the catalytic components therein, are highly conserved in sorghum and in rice PSTOL1 proteins (**Figure 1**). All PSTOL1 proteins start with the ATP-binding Site (BS in **Figure 1**), followed by the Active Site (AS) and the Activation Loop (Loop) and are all significantly variable with regards to extracellular domains. The size of the functional regions is remarkably identical across proteins, with the ATP-binding site comprised of 23 residues whereas the active site and the activation loop have 13 and 30 amino acid residues, respectively.

### 3.2 Sequence conservation of the PSTOL1 kinase core

**Figure 2:**
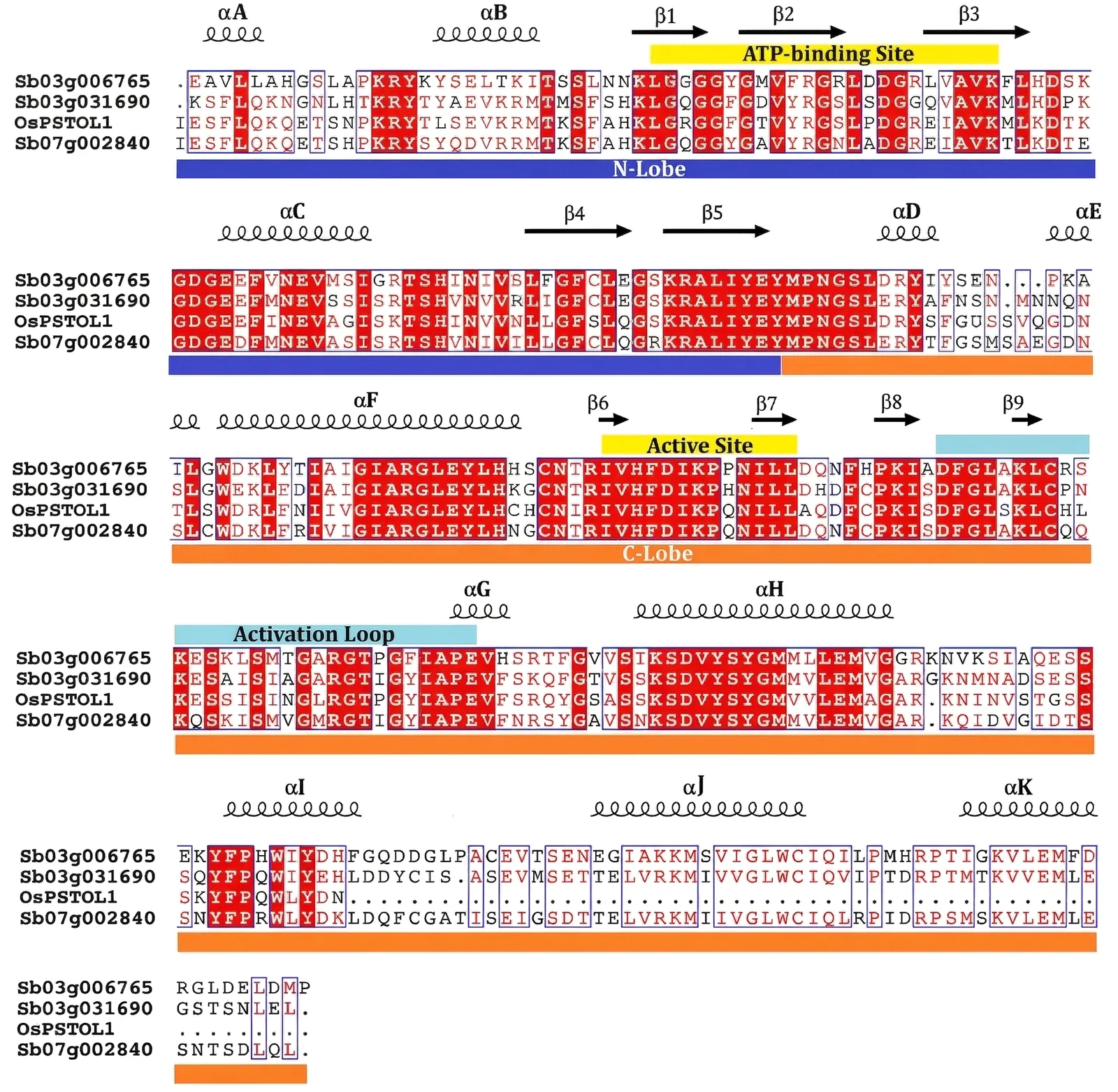
Multiple sequence alignment of PSTOL1 kinase domains in sorghum and rice. Residues in white letters on a red background are 100% identical among proteins whereas amino acids in red inside blue boxes have similar physicochemical properties. The N-lobe (blue bar underneath the sequence alignment) and the C-lobe (orange bar) are shown. The secondary structure elements, α-helices (αA-K) and β-strands (β1-9, indicated by arrows) are depicted above the sequence alignments. Horizontal bars on top show the conserved ATP-binding site, the active site (yellow) and the activation loop (cyan).

Multiple sequence alignment of the kinase domains revealed an exceptionally high degree of conservation between the PSTOL1 proteins of sorghum and rice. While the full-length amino acid sequences exhibit amino acid identity ranging from 44% to 72%, the isolated kinase domains have between 61% and 74% identity, with 100% coverage across all sequences (**Figure 2**).

The secondary structure elements and functional motifs in the kinase domain are conserved across sorghum and rice PSTOL1 proteins. For standardization, catalytic sites are defined herein as the essential regions for ATP coordination and phosphate transfer. Detailed analysis of these sites with respect to OsPSTOL1 indicated specific conservation patterns. The ATP-binding site shows identity between 58% and 71% and ends with four conserved residues within the β3-sheet. In Sb07g002840 and OsPSTOL1 this motif is IAVK, whereas Sb03g006765 and Sb03g031690 exhibit a VAVK variant (**Figure 2**). Fundamentally, the lysine (K) residue within these β3-strand motifs forms the canonical salt bridge (αC-Glu ↔ β3-Lys), which is essential for stabilizing the PSTOL1 catalytic sites.

The active site was remarkably conserved across PSTOL1 proteins, with sequence identity ranging from 92% to 100%. The Sb07g002840 sequence is identical to OsPSTOL1 in this region, whereas the other proteins differ by only a single amino acid across the 13-amino acid residue span. The motifs delimiting the activation loop, which show 70.0% and 76.7% sequence identity, are also strictly conserved, beginning with the DFGL motif and ending with the IAPE motif (**Figure 2**).

The spatial distribution of these sites within the kinase lobes is invariant: the ATP-binding site is located in the N-lobe whereas the active site and the activation loop are in the C-lobe (**Figure 2**). These results indicate that although divergence occurs in the extracellular domains, the structural core responsible for intracellular signaling remains highly conserved across PSTOL1 proteins in rice and sorghum.

To assess whether the extracellular domains and other conserved features identified in the SbPSTOL1 proteins are sorghum-specific or more broadly distributed in the Andropogoneae tribe, we retrieved and analyzed PSTOL1 orthologs from representative genera in the Andropogoneae, namely *Saccharum*, *Andropogon* and *Miscanthus* (**Supplementary Figures S2**, **S3**). Using a maximum likelihood-based phylogeny of the kinase domain, we show the relationship between PSTOL1 proteins from sorghum and rice and related orthologs in the Andropogoneae (**Supplementary Figure 2**). We found that the general protein domain architecture was largely conserved (**Supplementary Figure 3**). Most orthologous proteins retained the same extracellular GUB_WAK and WAK-associated domains together with the intracellular kinase domain, and all carried the conserved active and ATP-binding sites. *Miscanthus sinensis* (08G007500) and *Saccharum* R570 (08Cg037800) have only transmembrane domains and the later also has a LysM domain.

These results suggest that the extracellular domains identified in the SbPSTOL1 proteins are not sorghum-specific but are in fact substantially conserved across the Andropogoneae, though, once again, in a polymorphic fashion. Hence, the cytosolic architecture of OsPSTOL1 remains an exception. Consistent with this conservation, the catalytic-loop motif of the SbPSTOL1 proteins, the related ZmWAKL and OsPSTOL1 carries a non-arginine residue (an HFD-type motif) immediately preceding the catalytic aspartate, classifying them as non-RD kinases (Dardick and Ronald, 2006).

### 3.3 Structural modelling of PSTOL1 proteins

The structural prediction confidence for SbPSTOL1 monomers was evaluated locally using the Predicted Local Distance Difference Test (pLDDT) and globally by the Predicted Template Modeling score (pTM). pLDDT values above 70 indicate high-quality backbone prediction. Regarding pTM, models exceeding 0.5 have a high probability of exhibiting the correct topological fold, while values above 0.7 are considered of high confidence, hence adequate for functional inferences (Xu and Zhang, 2010; Jumper et al., 2021).

Results from AlphaFold2 and 3 displayed consistent mean pLDDT scores between 75 and 80, indicating that local secondary structure was predicted with high confidence (**Table 1**). However, inspection of pTM revealed differences between software versions, AlphaFold3 models yielded pTM values ranging from 0.53 to 0.61, consistent with an intermediate precision range. Conversely, pTM values for RoseTTAFold1 (~0.67) were consistently higher than that of AlphaFold3 (~0.57), indicating higher global confidence in SbPSTOL1 structure with the former.

**Table 1:** Confidence scores (metrics) of predicted SbPSTOL1 structures.

Software	Metrics	Sb03g006765	Sb03g031690	Sb07g002840
AlphaFold3	pTM	0.61	0.53	0.57
	pLDDT	76	77	75
AlphaFold2	pLDDT	77	80	78
RoseTTAFold2	pLDDT	66	67	69
RoseTTAFold-All-Atom	pLDDT	67	66	68
RoseTTAFold1	pTM	0.68	0.66	0.67

pLDDT is the Predicted Local Distance Difference Test and pTM is the Predicted Template Modeling score. Numbers following software names indicate software versions.

**Figure 3:**
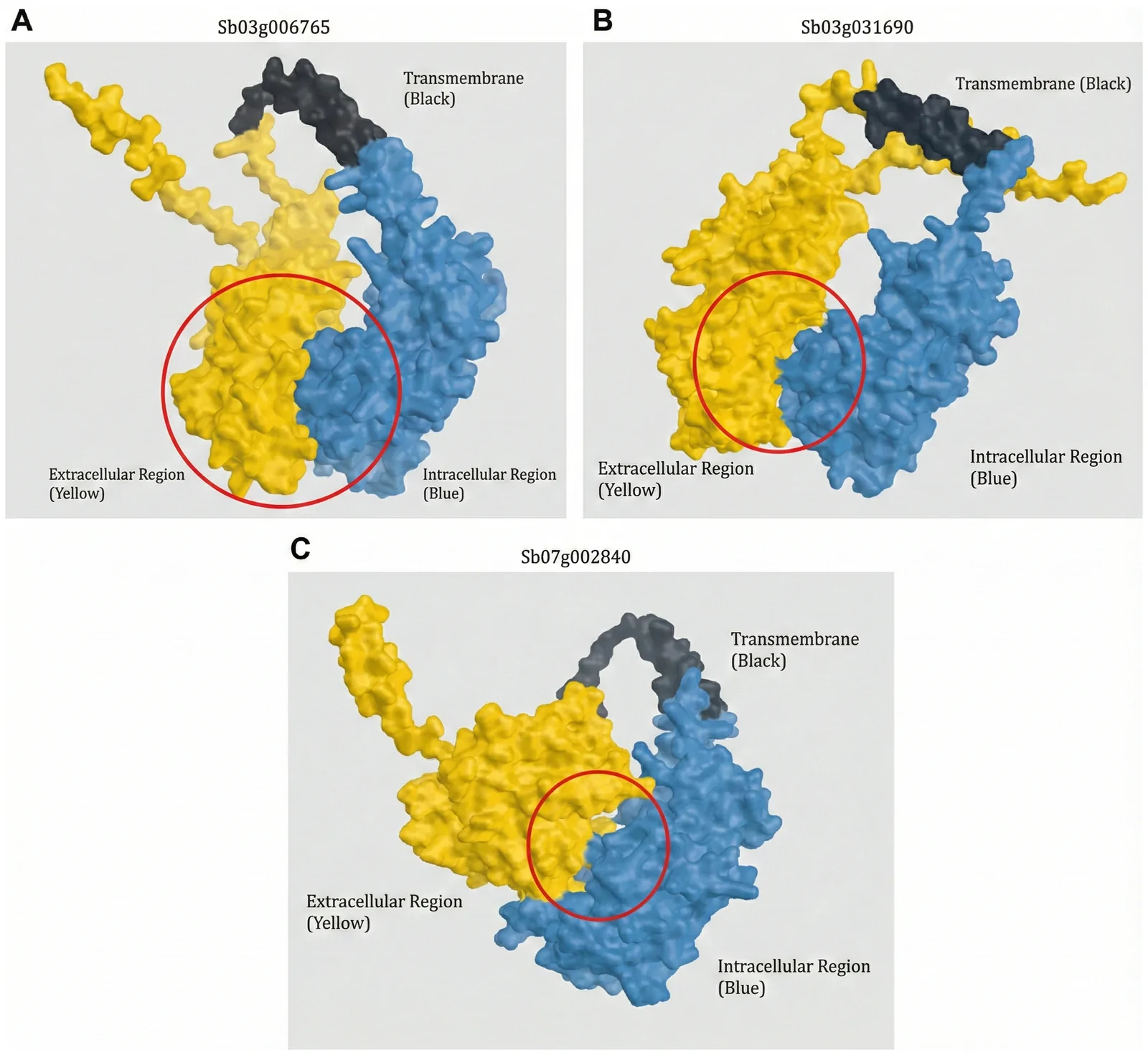
Monomeric structural models of SbPSTOL1 proteins predicted with AlphaFold3. Three-dimensional models of **(A)** Sb03g006765, **(B)** Sb03g031690 and **(C)** Sb07g002840. Extracellular, transmembrane and intracellular domains predicted with Interproscan are color coded. Red circles depict contacts deemed biologically inviable given the tripartite structure of plant RLKs. In all cases, a collapsed globular conformation that fails to maintain the spatial segregation between the external and internal protein portions was observed.

The monomeric structural predictions generated with AlphaFold2, AlphaFold3, RoseTTAFold2 and RoseTTAFold-All-Atom resulted in a systematic structural collapse across all SbPSTOL1 proteins (**Figure 3**). In these models, despite high local confidence scores, direct contacts and hence topological overlap were observed between the extracellular domains and the intracellular kinase domains (indicated by red circles in **Figure 3**). Furthermore, the single-pass transmembrane helices appear severely flexed and distorted, occasionally establishing spurious contacts with the kinase domain. This global configuration fails to maintain the rigorous spatial segregation between the external and internal portions of the protein that is required for a viable RLK architecture.

### 3.4 Domain organization and catalytic pocket accessibility in a membrane environment

**Figure 4:**
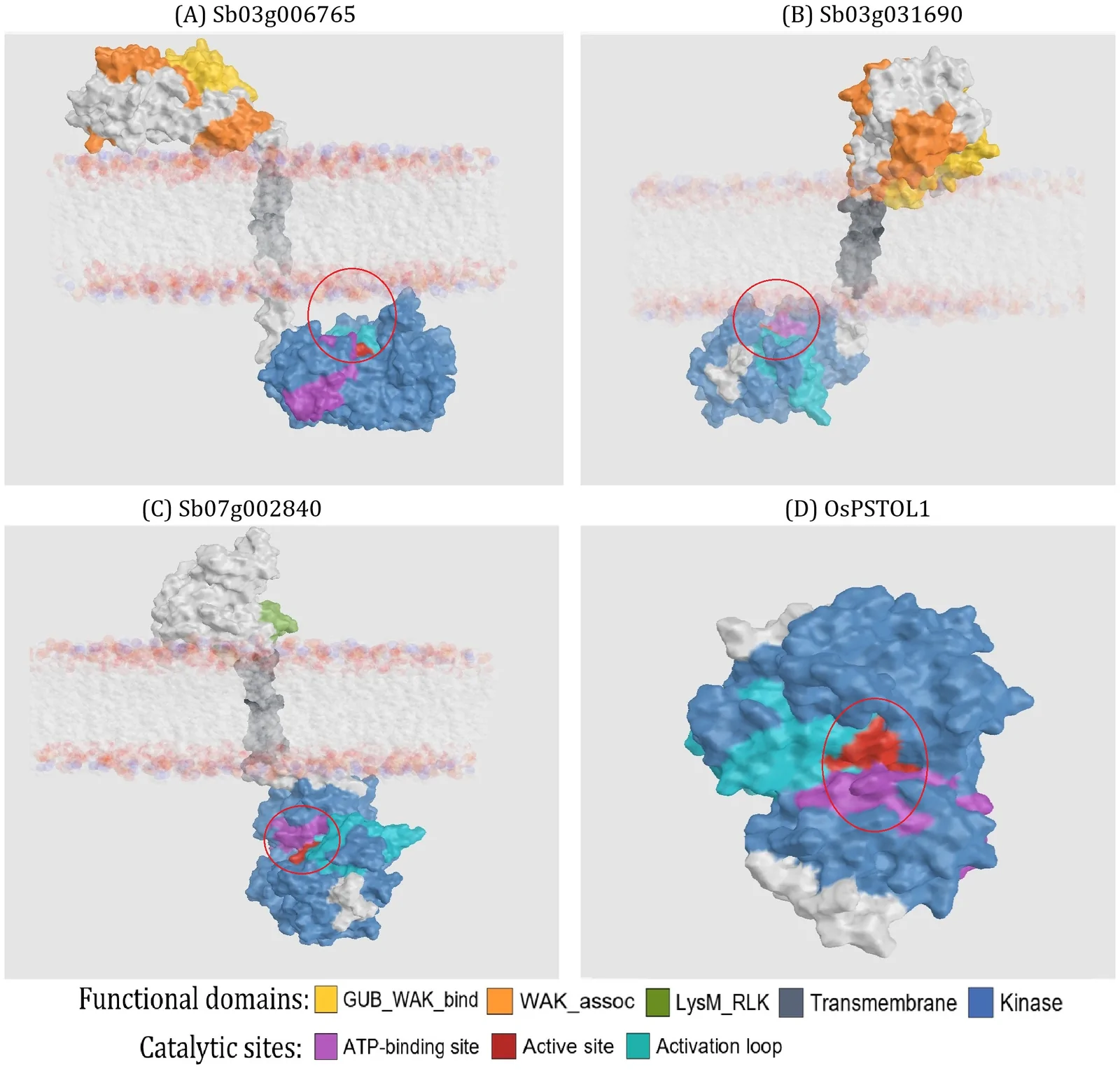
Structural organization and catalytic pocket of PSTOL1 proteins. Predicted 3D structures generated with RoseTTAFold1 for **(A)** Sb03g006765, **(B)** Sb03g031690, **(C)** Sb07g002840 and **(D)** OsPSTOL1. The proteins are shown embedded in a simulated POPC lipid bilayer and the domains, GUB_WAK_bind, WAK_assoc, LysM_RLK, transmembrane α-helix and kinase are color coded. Red circles highlight the catalytic pocket, formed by the spatial convergence of the ATP-binding site, active site and the activation loop.

RoseTTAFold1 stood out as the only software capable of establishing the expected tripartite architecture for the SbPSTOL1 proteins (**Figures 4A–C**). In contrast to the globular structure generated with other algorithms, which are likely modeling artifacts, the RoseTTAFold1 models displayed rigorous spatial domain segregation, correctly positioning the extracellular and intracellular domains on opposite faces of a single-pass transmembrane helix.

Bioinformatic analysis reinforces the viability of this topology, as the membrane insertion coordinates predicted with the PPM 2.0/OPM server (Lomize et al., 2011) showed an exact correspondence with the domain annotations obtained with InterProScan (Blum et al., 2020). These results show that the transmembrane helix likely traverses the lipid bilayer in a manner that projects the extracellular domains (WAK and LysM) into the apoplast. Such structural configuration is compatible with both physical anchoring to the cell wall and the perception of soluble ligands in the extracellular space, allowing the sensor domains to scan the environment for environmental signals (Kohorn and Kohorn, 2012; Whyte and Sipahi, 2023).

Visual inspection of the cytoplasmic kinase domain, supported by the 3D rotations in **Supplementary Videos S1–S4**, revealed critical variations in the accessibility of the catalytic pocket. In Sb03g006765 (**Figure 4A**; **Supplementary Video S1**) and Sb03g031690 (**Figure 4B**; **Supplementary Video S2**), a rotational tilt of the kinase domain is observed, directing the ATP-binding site (purple) and the active site (red) toward the inner leaflet of the membrane (the catalytic sites are depicted with a red circle in **Figure 4**). This positioning suggests potential plasma membrane steric occlusion of these catalytic sites, which serve as the primary contact points for cofactor recruitment and interaction with protein substrates during signaling (Hohmann et al., 2017).

In contrast, in Sb07g002840 (**Figure 4C**; **Supplementary Video S3**) and OsPSTOL1 (**Figure 4D**; **Supplementary Video S4**), the three catalytic sites, the ATP-binding site, the active site and the activation loop converge to form a solvent-accessible catalytic pocket. In these architectures, the spatial orientation of the activation loop does not obstruct the ATP-binding or active sites. This arrangement is maintained by the specific tilt of the kinase domain relative to the membrane, which likely keeps the catalytic pocket available for interaction with cytoplasmic substrates.

### 3.5 Structural modelling of ZmWAKL and ZmWIK

**Figure 5:**
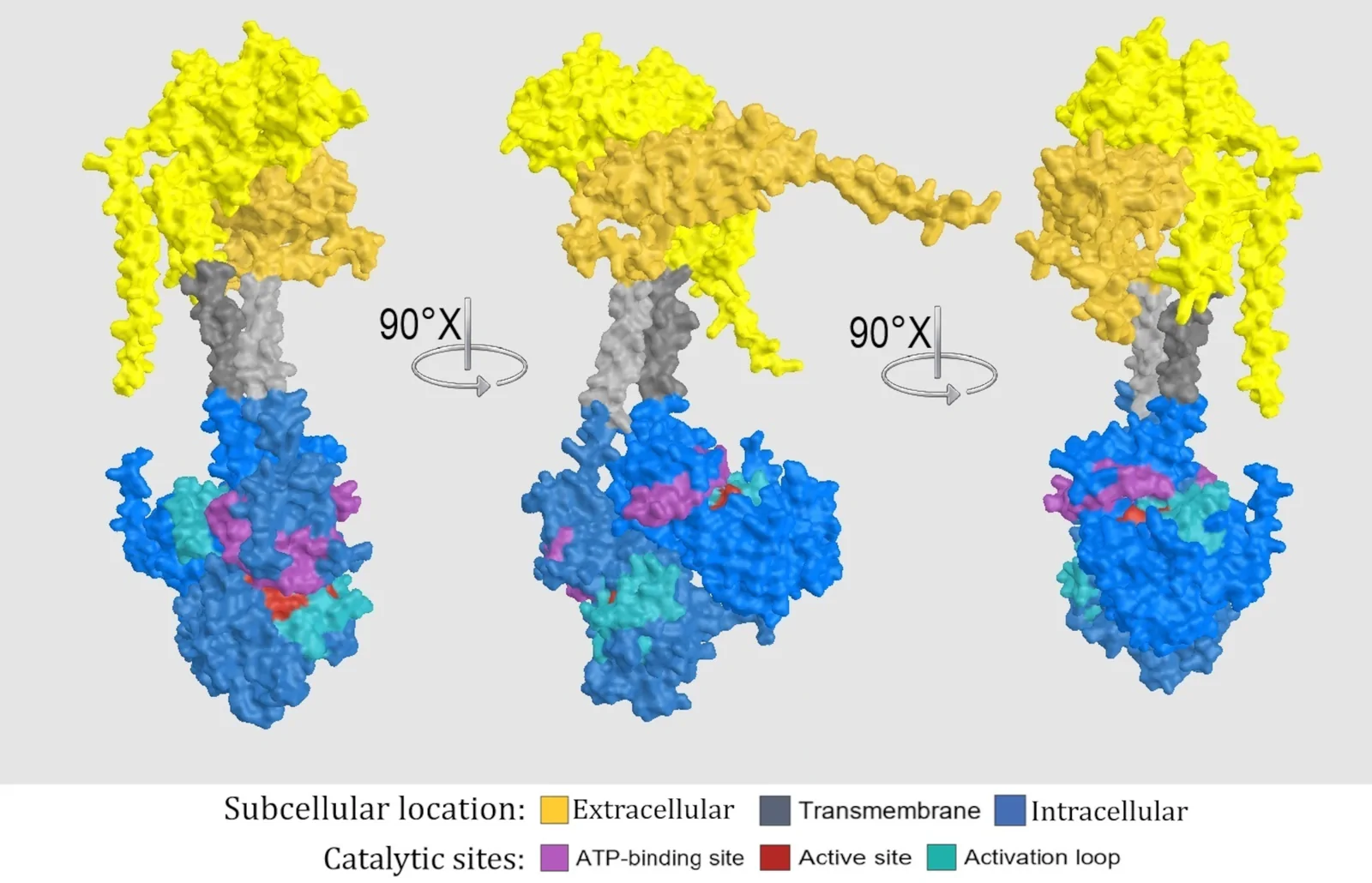
AlphaFold3-predicted structural model of the ZmWAKL–ZmWIK complex. The ZmWAKL–ZmWIK heterodimer is displayed in three views generated by successive 90° rotations about the vertical X-axis. Subcellular domain locations, namely the extracellular and intracellular domains and the single-pass transmembrane helix predicted with InterProScan (Blum et al., 2020) are color-coded. Specifically, stronger color tones indicate ZmWIK and lighter tones indicate ZmWAKL. Note the antiparallel “back-to-back” arrangement of the active site and the ATP-binding site of ZmWAKL, which are solvent-exposed and thus clearly visible in the left-most view, whereas the same catalytic sites of ZmWIK are visible in the middle and right-most views.

As an exploratory, initial step in RLK oligomer modeling, the ZmWAKL and ZmWIK monomers and their heterodimer (Zhong et al., 2024) were modelled with AlphaFold2, AlphaFold3, RoseTTAFold v1/v2 and RoseTTAFold-All-Atom, and the resulting models were examined for structural collapse by visual inspection in PyMOL. Contact analysis of viable models was performed with COCaDA. For the monomers, only RoseTTAFold v1 produced non-collapsed models of both ZmWAKL and ZmWIK, whereas AlphaFold3 produced a non-collapsed model of ZmWAKL but not of ZmWIK, which collapsed; the corresponding confidence scores are reported in **Supplementary Table S1**. For the heterodimer, only AlphaFold3 generated a model without structural collapse. As shown in **Figure 5**, AlphaFold3 produced a ZmWAKL–ZmWIK complex exhibiting rigorous spatial segregation between the extracellular and cytoplasmic portions, driven by the transmembrane domains, whereas all the other tools showed structural collapse.

Contact analysis with COCaDA indicated that the predicted AlphaFold3 interface spans the extracellular, transmembrane and intracellular regions simultaneously, consistent with the experimentally mapped interaction (Zhong et al., 2024). These results further reinforce that, among the tools tested, AlphaFold3 provides the prediction most consistent with the known ZmWAKL – ZmWIK interaction (**Figure 5**). At the dimer interface, COCaDA identified 131 inter-chain contacts (6 attractive, 1 aromatic-stacking, 36 hydrogen bonds, 78 hydrophobic, 5 repulsive and 5 salt bridges), distributed across the extracellular (40), transmembrane (10) and intracellular (81) regions. Three of these contacts involved residue 512R of the ZmWAKL activation loop with ZmWIK, whereas neither the active site nor the ATP-binding site of ZmWAKL contacted ZmWIK, and none of the catalytic sites of ZmWIK contacted ZmWAKL. These results indicate that the predicted interface leaves both catalytic pockets in ZmWAKL – ZmWIK solvent-exposed. Notably, ZmWAKL is a non-RD kinase, a feature shared with the SbPSTOL1 proteins and also with rice OsPSTOL1. A detailed analysis of the intracellular portion of the ZmWAKL–ZmWIK heterodimer revealed that the kinase domains are organized in an antiparallel geometry, usually described as a “back-to-back” configuration (Bojar et al., 2014; Hohmann et al., 2018). This arrangement positions the catalytic clefts of both monomers on opposite faces (see 90° rotations in **Figure 5**), suggesting that the catalytic pocket of each protein may remain exposed to the solvent and accessible for substrate binding and ATP-Mg^2+^ complex formation. The precise reproduction of this geometry, which is validated by established literature on RLK allosteric activation (Bojar et al., 2014; Hohmann et al., 2018), provides the necessary methodological support for modeling SbPSTOL1 oligomeric complexes.

### 3.6 Structural prediction and interface analysis of SbPSTOL1 oligomeric complexes

**Figure 6:**
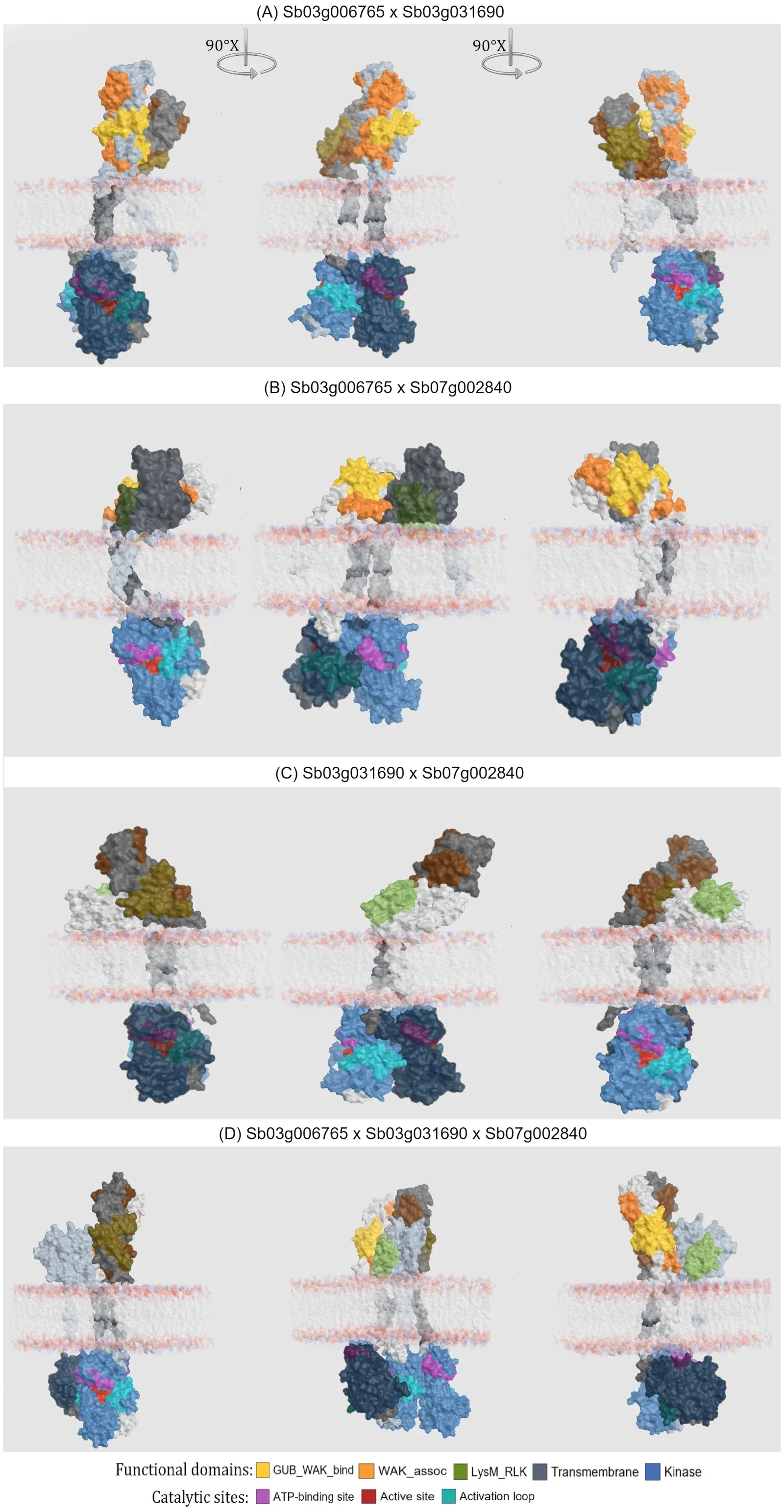
Structural modeling and site accessibility of SbPSTOL1 oligomeric complexes. AlphaFold3-predicted 3D structures embedded in a simulated plasma membrane. Each panel displays a protein complex in three orthogonal views (produced with 90° rotations around the X-axis) to demonstrate domain organization. **(A)** Sb03g006765 x Sb03g031690, **(B)** Sb03g006765 x Sb07g002840 and **(C)** Sb03g031690 x Sb07g002840 show dimers with catalytic clefts exposed to the cytosol in an antiparallel orientation. **(D)** The heterotrimer reveals spatial confinement of the Sb03g031690 and Sb07g002840 catalytic sites due to the face-to-face orientation of the kinase domains, obstructing the catalytic pocket of both proteins. To facilitate protein identification in the oligomers, one partner in each complex is represented with a darker surface shading. Specifically, Sb07g002840 is identified by the presence of the LysM domain (green). In oligomers containing both Sb03g006765 and Sb03g031690, the latter is consistently represented by the darker shaded surface. The extracellular and the kinase domains, in addition to the transmembrane domains, are color-coded. Rotational animations demonstrating site exposure are shown in **Supplementary Videos S5–S8**, with Video S8 showing the heterotrimer.

In oligomeric models predicted with AlphaFold3, the transmembrane regions of each partner protein remained parallel to each other and correctly inserted into the plasma membrane plane, with no evidence of globular collapse (**Figure 6**). Visual analysis of the cytoplasmic interfaces of SbPSTOL1 oligomers suggested that complex formation may induce a conformational shift, where the catalytic clefts, comprising the ATP-binding site, the active site and the activation loop transitioned from a membrane-occluded configuration in the monomers to a solvent-accessible geometry in the oligomers (**Supplementary Videos S5–8**). Similar to ZmWAKL-ZmWIK, in the SbPSTOL1 heterodimers (**Figures 6A–C**; **Supplementary Videos S5–7**), the kinase domains are organized in an antiparallel (back-to-back) geometry, positioning the functional pockets on opposite faces of the complex. An interatomic contact analysis was subsequently conducted using the COCaDA software (Lemos et al., 2025) and direct interactions between the active site and the ATP-binding site residues in SbPSTOL1 monomers were absent (**Figures 4A–D**; **Supplementary Videos S1–S4**). This supports the AlphaFold3 model, indicating that the catalytic pocket of the SbPSTOL1 heterodimers (**Figure 6**) is apparently free of interfacial obstructions, which is independently supported by the interface analysis done with COCaDA (**Supplementary Table S2**).

Regarding the SbPSTOL1 interfaces (**Supplementary Table S2**), stabilization is predominantly driven by intracellular interactions between the kinase domains, with contacts ranging from 78 (Sb03g006765 × Sb03g031690) to 97 (Sb03g031690 × Sb07g002840). However, distinct patterns were observed in the extracellular domains. In the Sb03g006765 × Sb03g031690 heterodimer (**Figure 6A**), the ECDs exhibit a pronounced vertical orientation away from the plasma membrane. The extracellular interface involves contact between residues 113R in the GUB_WAK domain of Sb03g006765 and 267E in the WAK_Assoc domain of Sb03g031690, which establish one salt bridge and four hydrogen bonds. In the Sb03g006765 × Sb07g002840 heterodimer (**Figure 6B**), the complex displays an extracellular interface with 55 contacts involving 16 residues from each monomer (**Supplementary Table S2**). Notably, no residues from the LysM-RLK domain of Sb07g002840 participate in the interface, which is primarily composed of hydrophobic interactions and hydrogen bonds.

The Sb03g031690 × Sb07g002840 heterodimer (**Figure 6C**) shows 22 extracellular contacts involving the GUB_WAK and WAK_Assoc domain of Sb03g031690 (**Supplementary Table S2**). Similarly, the LysM-RLK domain of Sb07g002840 establishes no interfacial contacts. In the Sb03g006765 × Sb03g031690 × Sb07g002840 heterotrimer (**Figure 6D**), the contact topology undergoes a drastic alteration. The Sb03g006765 protein acts as a central scaffold, maintaining 91 kinase-domain contacts with Sb07g002840. In contrast, while 10 extracellular contacts were detected between Sb03g006765 and Sb03g031690, no intracellular interactions between these SbPSTOL1 proteins were detected. COCaDA did not detect any contacts between the extracellular regions of Sb03g006765 or Sb03g031690 with Sb07g002840. The interface between Sb03g031690 and Sb07g002840 is solely intracellular, with 44 contacts between their kinase domains.

Although interface analysis with COCaDA did not detect direct inter-chain contacts within the active or ATP-binding sites, it did reveal critical interactions localized specifically within the activation loops of all studied complexes. In the Sb03g006765 × Sb03g031690 heterodimer, it is noteworthy that, at the interface specifically between the activation loops, the interacting residues involved are 469K (Sb03g006765) and 512K (Sb03g031690). A similar pattern of stabilization via hydrogen bonds was observed in the Sb03g031690 × Sb07g002840 heterodimer, involving residues 512K and 470K, respectively. In contrast, the Sb03g006765 × Sb07g002840 complex exhibited a non-reciprocal interface, where only the Sb07g002840 activation loop established a hydrogen bond of amino acid residues, 470K with 452N (Sb03g006765), while Sb03g006765 lacked interfacial contacts within its activation loop. Generally, activation loop interfaces in SbPSTOL1 complexes are mediated by the conserved residues 469K (Sb03g006765), 512K (Sb03g031690), and 470K (Sb07g002840). This involvement of the activation loop in the interface is consistent with the canonical allosteric activation model of plant RLKs, such as the BRI1–SERK complex, where cytosolic kinase proximity acts as the molecular trigger for signal initiation (Hohmann et al., 2017).

However, in the heterotrimer, interfacial complexity increases significantly (**Supplementary Table S2**), and the Sb03g031690 and Sb07g002840 subunits do not follow this specific contact pattern. While Sb03g006765 maintains its contact via 469K with Sb07g002840, the Sb03g031690 and Sb07g002840 subunits establish a robust reciprocal interface featuring 13 and 23 contacts, respectively, which are predominantly hydrophobic. Visual inspection reveals that the kinase domains of Sb03g031690 and Sb07g002840 adopt a face-to-face orientation, resulting in steric hindrance and hence likely occlusion of their catalytic pockets. Conversely, the catalytic pocket of the Sb03g006765 subunit remains fully exposed to the cytosol, suggesting that the complex topology possibly influences signaling selectivity.

### 3.7 Analysis of the conserved αC-Glu ↔ β3-lys salt bridge in SbPSTOL1 proteins

The structural compatibility with catalytic competence of SbPSTOL1 was assessed by studying structural integrity of their functional core, specifically monitoring the conserved electrostatic interaction between the glutamate residue (E) in the αC-helix and the lysine (K) in the β3-strand. This so-called salt bridge acts as a critical “molecular switch”, whereby the αC-helix is repositioned inward with regards to the active site (αC-in), enabling the glutamate residue in the αC-helix and the lysine in the β3-strand of the N-lobe to align, correctly coordinating ATP phosphates for the phosphoryl transfer reaction (Taylor and Kornev, 2011).

**Figure 7:**
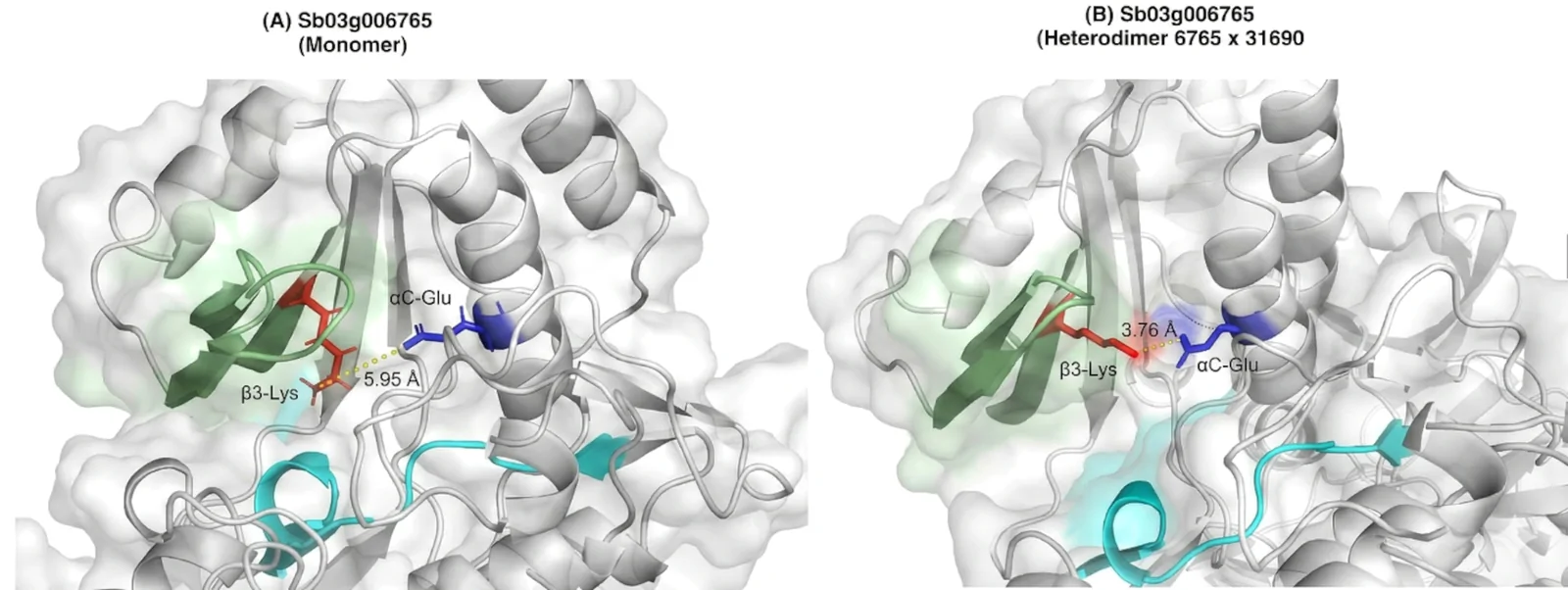
Spatial orientation and integrity of the catalytic salt bridge in Sb03g006765. **(A)** Inactive state. Detailed view of the catalytic site in the Sb03g006765 monomer, where a distance of 5.95 Å between the misaligned αC-Glu and β3-Lys residues exceeds the salt bridge formation threshold. **(B)** Active configuration. The distance between the αC-Glu and β3-Lys residues in the Sb03g006765 x Sb03g031690 heterodimer is now reduced to 3.76 Å The ATP-binding site and the active site are shown in green and cyan, respectively.

**Table 2** shows that the αC-Glu ↔ β3-Lys distance is compatible with salt bridge formation (below 4 Å) only in OsPSTOL1 and in the SbPSTOL1 oligomers (3.33 Å to 4.00 Å), but not in any of the SbPSTOL1 monomers, which show distances between 5.37 Å to 5.95 Å. These results indicate that the transition to an active conformation is dependent on the oligomerization state of the SbPSTOL1 proteins. For OsPSTOL1, the literature suggests additional regulatory mechanisms, such as SUMOylation, which is required for its kinase activity (Mukkawar et al., 2024). As illustrated in **Figure 7A** for the Sb03g006765 monomer, a 5.95 Å separation between the β3-Lys (red) and αC-Glu (blue) residues results in a structurally misaligned ATP-binding site, characteristic of an inactive state. This behavior contrasts with that of the rice protein OsPSTOL1, a cytosolic kinase whose structure is compatible with an inherently formed salt bridge regardless of its monomeric state.

**Table 2:** Analysis of the salt bridge distance (αC-Glu ↔ β3-Lys).

Protein	Oligomer	β3-Lys	αC-Glu	Distance (Å)	Activation status
OsPSTOL1	monomer	K67	E82	3.57	active
Sb03g006765	monomer	K345	E360	5.95	inactive
Sb03g031690	monomer	K386	E401	5.77	inactive
Sb07g002840	monomer	K343	E358	5.37	inactive
Sb03g006765	6765 x 31690	K345	E360	3.76	active
Sb03g031690	6765 x 31690	K386	E401	3.50	active
Sb03g006765	6765 x 2840	K345	E360	3.75	active
Sb07g002840	6765 x 2840	K343	E358	3.70	active
Sb03g031690	31690 x 2840	K386	E401	3.75	active
Sb07g002840	31690 x 2840	K343	E358	4.00	active
Sb03g006765	6765 x 31690 x 2840	K345	E360	3.79	active
Sb03g031690	6765 x 31690 x 2840	K386	E401	3.33	active
Sb07g002840	6765 x 31690 x 2840	K343	E358	3.39	active

The inferred activation status indicates whether a salt bridge is formed (active) or broken (inactive) based on the distance between the αC-Glu and β3-Lys residues. The threshold for salt bridge formation is based on the interaction standards established by Jones and Thornton (1996) and the geometric criteria implemented in the COCaDA software (Lemos et al., 2025). The “active” configuration is functionally consistent with the kinase activation mechanism described by Taylor and Kornev (2011).

The interaction between Sb03g006765 and Sb03g031690 is primarily driven by specific hydrogen bonds between the activation loop residues, 469K and 512K. This inter-monomer contact serves as a structural trigger associated with a conformational transition of the kinase domain. Consequently, this conformational shift reduces the distance between the αC-Glu and β3-Lys catalytic residues in Sb03g006765 from 5.95 Å in the monomeric state to 3.76 Å in the heterodimer. This transition is consistent with the formation of a functional salt bridge and a catalytically competent cleft only within the SbPSTOL1 heterodimeric context. Furthermore, visual inspection suggests that this oligomerization-induced movement decreases the steric obstruction of the active site relative to the inactive monomer, potentially facilitating greater accessibility for the catalytic machinery.

This activation trend is even more pronounced in the heterotrimer, where all three SbPSTOL1 partners reached the active state simultaneously, resulting in the shortest distances between the αC-Glu and β3-Lys residues, of 3.79 Å for Sb03g006765, 3.33 Å for Sb03g031690 and 3.39 Å for Sb07g002840 (**Table 2**).

### 3.8 Molecular dynamics of SbPSTOL1 proteins

We simulated how the SbPSTOL1 structure embedded in a hydrated membrane environment changes over time by means of a molecular dynamics approach, whereby individual atoms are positionally moved in a type-dependent way. By assessing whether a given motion is physically consistent with the system potential energy, mathematically represented by the force field, this approach attempts to reproduce protein structure in the context of the cell environment, which is influenced by factors like temperature, pressure and chemical conditions (Phillips et al., 2020). First, we measured convergence of each system, defined as the time-point where RMSD reaches stability. The trajectory times were 300 nanoseconds (ns) for the Sb03g006765 × Sb03g031690 heterodimer, 125 ns for Sb03g031690 × Sb07g002840 and approximately 100 ns and 60 ns for the Sb03g006765 × Sb07g002840 heterodimer and the heterotrimer, respectively.

**Figure 8:**
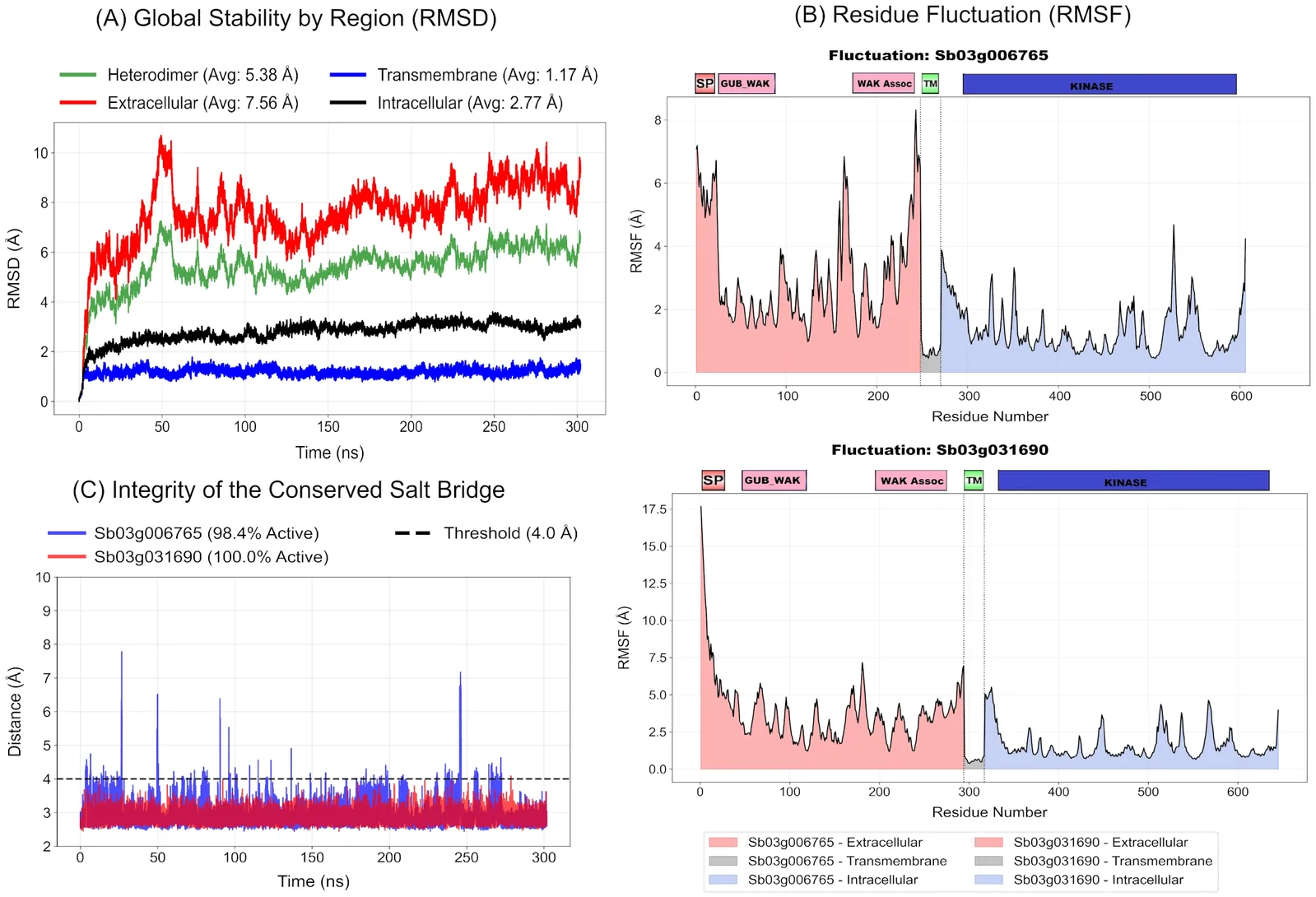
Molecular dynamics and distance-compatible salt bridges in Sb03g006765 × Sb03g031690. **(A)** Global stability by region measured by Root Mean Square Deviation (RMSD) trajectories over a 300 ns simulation for the heterodimer, transmembrane domain (TM), intracellular domain (kinase), and extracellular domain. **(B)** Root Mean Square Fluctuation (RMSF) trajectories, with individual protein domains identified by horizontal bars above each graph. **(C)** Integrity of the conserved salt bridge monitored by the distance between αC-Glu ↔ β3-Lys over time. The interaction remains consistently below the threshold of 4.0 Å (dashed line). SP, signal peptide.

The structural integrity of the SbPSTOL1 complexes was supported by the convergence of RMSD to a stable plateau for the intracellular regions of the oligomers, which remained below the 3.0 Å threshold for all heterodimers and reached 3.25 Å for the heterotrimer (**Table 3**). This distance range is widely recognized as indicative of a preserved native fold for proteins of similar sizes (Maiorov and Crippen, 1994; Martínez, 2015). No evidence of denaturation was observed, as the systems did not exhibit the characteristic increasing RMSD drift or the large-scale structural expansions (> 10 Å) associated with protein unfolding. The results for the Sb03g006765 × Sb03g031690 complex are shown in **Figure 8**, while the trajectories for the additional heterodimers and the heterotrimer are presented in **Supplementary Figures S4–S6**.

**Table 3:** Root mean squared deviation (RMSD) (Å) for SbPSTOL1 oligomers by region and for entire oligomers.

	
SbPSTOL1 Oligomers	Oligomer	Extracellular	TM	Intracellular
Sb03g006765xSb03g031690	5.38	7.56	1.17	2.77
Sb03g006765xSb07g002840	5.06	7.05	1.24	3.09
Sb03g031690xSb07g002840	4.22	5.53	0.69	2.93
Heterotrimer	4.12	5.14	0.90	3.25

The heterotrimer is comprised of the SbPSTOL1 proteins, Sb03g006765, Sb03g031690 and Sb07g002840. Each oligomer had distinct trajectory times, as described previously. TM: α-helix transmembrane domain.

The RMSD trajectories (**Figure 8A**) indicate that the Sb03g006765 × Sb03g031690 heterodimer reaches structural equilibrium, with values remaining within a stable range between 5 and 7 Å throughout the 300 ns simulation. This stability was found to be strongly domain-dependent; while the transmembrane and intracellular kinase regions stabilized remarkably early, at 1 ns and 25 ns, respectively, with low mean RMSD values of 1.17 Å and 2.77 Å (**Table 3**), the extracellular domain exhibited a significantly higher mean RMSD of 7.56 Å. Notably, the overall mobility of the heterodimer closely mirrors the high flexibility of its extracellular regions, suggesting that the TM domain functions as a mechanical insulator (**Supplementary Figures 4–6**).

The Root Mean Square Fluctuation (RMSF) profiles (**Figure 8B**) are consistent with the stability of the intracellular catalytic core, with fluctuations confined between 1.4 Å and 1.8 Å. The highest mobility peaks were again observed for the extracellular regions, specifically within the GUB_WAK and WAK_Assoc domains, where fluctuations exceeded 6.0 Å, and also for the signal peptides. Structural compatibility with catalytic competence maintenance was monitored by the distance trajectory of the αC-Glu ↔ β3-Lys salt bridge (**Figure 8C**). In the Sb03g006765 × Sb03g031690 heterodimer, the salt bridge remained active, below the 4.0 Å threshold, for more than 97% of the simulation time. High stability of the salt bridge was also observed in the heterotrimer and in the Sb03g031690 × Sb07g002840 heterodimer (**Supplementary Figure 4**), with the distances between αC-Glu and β3-Lys remaining below 4.0 Å for 97% and 100% of the simulation time. The Sb03g006765 × Sb07g002840 heterodimer exhibited a distinct stability pattern of the salt bridge, with much stronger fluctuations in RMSD over time (**Supplementary Figure 5**). During the 100 ns analysis, the salt bridge remained active for only 28.1% of the time for Sb07g002840 and 61.7% for Sb03g006765.

## 4 Discussion

Receptor-like kinases are fundamental post-translational regulators in numerous signaling pathways, playing critical roles in both biotic and abiotic stress tolerance (Liu et al., 2024). Belonging to the LRK10 subfamily, PSTOL1 proteins have been associated with enhanced grain yield for important staple food crops cultivated in soils with low P availability (Gamuyao et al., 2012; Hufnagel et al., 2014). Nevertheless, the physiological mechanism by which PSTOL1 proteins modulate root growth remains a significant knowledge gap, which can be narrowed down by a better understanding of the tertiary and quaternary structures of these proteins. This gap is compounded by the inherent difficulties in determining interactions of bitopic, single-pass membrane-bound RLK proteins which, with their complex topology, interact with the plasma membrane and occasionally simultaneously with the cell wall. Such interactions interfere with the accessibility required for molecular interactions (Hohmann et al., 2017) that are furthermore frequently weak, transient and dependent of extracellular perception of rarely known ligands. In addition, as an additional layer of difficulty, RLK proteins may interact with partners with different topologies, including those harboring multiple transmembrane helices (Hegedűs et al., 2022).

Although the Protein Data Bank (PDB) contains tens of thousands of entries, only 3.2% of the experimentally determined structures in PDB correspond to membrane-bound proteins, with a mere 0.23% representing bitopic proteins (figures of March 2026), like SbPSTOL1. Because modern, AI-driven algorithms that predict protein structures heavily rely on the PDB, we sought to investigate the efficiency at which these procedures are able to model the structure of RLK proteins such as SbPSTOL1. In doing so, we aimed at identifying structural features possibly linked to catalysis, paving the way for future application of these tools as guides to identify key components in the signaling pathway(s) whereby SbPSTOL1 modulate root growth, P acquisition and sorghum yields on soils with low P availability (Hufnagel et al., 2014; Bernardino et al., 2019; Bernardino et al., 2021; Maciel et al., in preparation).

Our first relevant finding was a clear disconnect between confidence scores commonly used to assess modelling accuracy by widely used tools like AlphaFold and RoseTTAFold and the predicted tripartite topology of SbPSTOL1 proteins. SbPSTOL1 models generated with AlphaFold2 and 3, although showing high local confidence scores (pLDDT scores between 75–80), systematically produced topologically inconsistent or collapsed SbPSTOL1 models, characterized by spurious contacts between intracellular and extracellular domains. One likely reason is that residue-based scores such as pLDDT often fail to account for the steric constraints posed by lipid bilayers, allowing severe topological errors to coexist with high confidence scores (Hegedűs et al., 2022), which is what we observed for SbPSTOL1 and even for known complexes like ZmWAKL–ZmWIK (Zhong et al., 2024). Although AlphaFold3 reproduced the heterodimeric architecture and domain segregation of ZmWAKL–ZmWIK, the resulting ipTM score was found to fall near or slightly below the standard 0.5 ipTM threshold frequently used to define high-confidence protein-protein interactions (Dunbrack, 2025; Varga et al., 2025). Hence, we conclude that, as proposed by Jumper et al. (2021), protein modelling efforts need to transcend the information conveyed by confidence scores and must necessarily integrate knowledge of protein topology and the conservation of functional motifs.

Upon modelling SbPSTOL1 proteins in a monomeric fashion, we found strikingly different results, depending not only on the algorithm used but also on the version of each tool. First, the optimized attention mechanism of AlphaFold that treats global conformation as a unified folding event (Jumper et al., 2021) introduced a systemic bias toward hydrophobic collapse of SbPSTOL1 proteins. Hence, we found that AlphaFold models were particularly sensitive to training with PDB, where protein structures are predominantly solved in aqueous- or detergent-based environments. In the AlphaFold monomeric models, the hydrophobic transmembrane helix of SbPSTOL1 was likely forcedly buried within the protein’s globular domains to minimize solvation potential (Hegedűs et al., 2022), resulting in collapsed structures.

RoseTTAFold1 was the only tool that produced monomeric SbPSTOL1 models consistent with the SbPSTOL1 tripartite topology. Interestingly, more recent versions of this tool, such as RoseTTAFold2 and RoseTTAFold-All-Atom, also produced inconsistent SbPSTOL1 models, similar to those generated with AlphaFold. While the RoseTTAFold1 algorithm iteratively takes into account the primary amino acid sequence, pairwise residue distances and the three-dimensional protein structure, these 3D tracks operate on 3D backbone coordinates (Baek et al., 2021). In contrast, 3D tracking in later models such as in RoseTTAFold-All-Atom operate at full atomic resolution (Krishna et al., 2024). Yet, the backbone procedure in RoseTTAFold1, where 3D tracking provides a geometric constraint that is updated simultaneously with the 2D maps, likely prevented the algorithm from “over-folding” the SbPSTOL1 hydrophobic domains, preserving the tripartite topology.

Although RosettaFold1 was able to model the structure of SbPSTOL1 monomers, an active salt bridge was absent in all monomers, indicating that, as it is typical in RLK proteins, dimerization is likely needed for SbPSTOL1 function. The SbPSTOL1 proteins studied here show complementary subcellular localizations, with Sb07g002840 and Sb03g006765 being anchored to the plasma membrane whereas Sb03g031690 is located both in the plasma membrane and in the cell wall (Maciel et al., in preparation). Previously, we showed that SbPSTOL1 proteins control similar, root-related phenotypes, for example root diameter and root surface area, under control of their native promoters (Hufnagel et al., 2014). In addition, SbPSTOL1 proteins enhance root growth rates in tobacco overexpression lines (Maciel et al., in preparation). These aspects hint at a concerted action of SbPSTOL1 proteins in enhancing P acquisition via modulation of root system morphology and architecture (Hufnagel et al., 2014). Because dimerization is common and an often-important component in RLK function (Hohmann et al., 2017) and protein-protein interaction predictions often reveal critical biological features and structural conformations that remain hidden or disordered in monomeric states (Humphreys et al., 2021b), we set out to investigate the structural plausibility of dimer formation among the three closely related SbPSTOL1 proteins.

A viable homodimeric model was only observed for Sb03g031690, suggesting that heterodimerization is a relevant feature of SbPSTOL1 function. Indeed, unlike the collapsed monomer predictions generated with AlphaFold, topologically consistent SbPSTOL1 oligomeric models were predicted with AlphaFold3, which also reproduced the heterodimeric architecture and domain segregation of the known ZmWAKL–ZmWIK complex (Zhong et al., 2024).

A paradigm of the RLK activation mechanism is the interaction between the BRI1 (BRASSINOSTEROID INSENSITIVE 1) kinase and its co-receptor SERK (SOMATIC EMBRYOGENESIS RECEPTOR KINASE), where dimerization positions the intracellular active sites in direct proximity, triggering reciprocal trans-phosphorylation and inter-activation (Bojar et al., 2014). Structurally, this activation depends on an oligomerization-induced rotational shift in the transmembrane helices, which enables proper alignment of the N- and C-lobes and effective ATP coordination (Hohmann et al., 2017). Importantly, from a functional standpoint, the SbPSTOL1 oligomeric models appear broadly compatible with features of the classical RLK activation model. However, SbPSTOL1 (like OsPSTOL1 and ZmWAKL) are non-RD kinases (Dardick and Ronald, 2006), whose protein members typically lack the conserved arginine coupling activation-loop phosphorylation to catalytic activation and are therefore often not regulated by the canonical activation-loop-phosphorylation mechanism of RD kinases (Johnson et al., 1996; Dardick et al., 2012).

The SbPSTOL1 interfaces were found to be primarily stabilized by a rigid conserved hydrophobic core in the intracellular kinase domain. A tightly packed hydrophobic core stabilizing kinase domains is consistent with general principles of globular protein structure (Jones and Thornton, 1996). In the case of SbPSTOL1, this stabilization was found to be mediated by specific contacts within the activation loops, predominantly driven by the conserved lysine residues, 469 in Sb03g006765, 512 in Sb03g031690 and 470 in Sb07g002840 (second lysine, K, in the activation loop, **Figure 2**). This suggests a critical functional role of these residues in stabilizing the hydrogen bonds required for correct αC-helix orientation. Lysine residues have been found to act as hydrogen bond donors that “lock” the loop into a functional conformation, facilitating the transition to the “αC-in” state and the subsequent formation of the catalytic salt bridge, αC-Glu ↔ β3-Lys (Taylor and Kornev, 2011; Endicott et al., 2012). The OsPSTOL1 structure is compatible with a formed salt bridge even as a monomer, which is consistent with its cytoplasmic nature. In turn, the catalytic competence of sorghum homologs appears to be dependent on oligomerization to reverse the autoinhibited state characterized by inactive salt bridges (Lemmon and Schlessinger, 2010).

Molecular dynamics simulations allowed us to study the persistence of oligomeric architectures in a simulated membrane environment (**Supplementary Video S9**). The structural integrity of the SbPSTOL1 complexes was supported by the convergence of RMSD to a stable plateau for the intracellular regions of the oligomers, which remained below the 3.1 Å threshold for all heterodimers and reached 3.25 Å for the heterotrimer (**Table 3**). This range is widely recognized as indicative of a preserved native fold for proteins of similar sizes (Maiorov and Crippen, 1994; Martínez, 2015). The slight deviation observed for the heterotrimer is consistent with the increased structural complexity and greater flexibility inherent in a three-partner assembly (Hospital et al., 2015), whereas the achievement of a stable plateau supports the overall stability of the system (Martínez, 2015). No evidence of denaturation was observed, as the complexes did not exhibit the characteristic increasing RMSD drift or the large-scale structural expansions (> 10 Å) associated with protein unfolding (Hospital et al., 2015).

Regional analysis suggested that structural stability of the SbPSTOL1 protein is largely domain-dependent. While the transmembrane and kinase domains exhibited exceptional rigidity, the extracellular regions and the signal peptide were highly flexible, with fluctuations frequently exceeding 6.0 Å. This behavior suggests that the transmembrane domain functions as a mechanical insulator, effectively protecting the positioning and orientation of the signaling machinery in the cytosol from stochastic fluctuations of the extracellular environment (Li and Hristova, 2006).

A potential contribution of oligomerization to an active-like SbPSTOL1 configuration is suggested by the stability of the canonical αC-Glu ↔ β3-Lys salt bridge (Taylor and Kornev, 2011; Endicott et al., 2012) along the simulated pathways. In the heterodimers Sb03g006765 × Sb03g031690 and Sb03g031690 × Sb07g002840, as well as in the heterotrimer, the salt bridge remained in an active configuration for more than 97% of the simulation time. In contrast, molecular dynamics revealed a critical instability in the Sb03g006765 × Sb07g002840 heterodimer, where the salt bridge failed to remain stable in both partners. This instability is likely explained by the absence of reciprocal contacts in the activation loop of the Sb03g006765 subunit, although such contacts are present in the Sb07g002840 loop. Since reciprocal contacts in the activation loop are consistently observed in all monomers forming stable oligomers and are described as necessary structural triggers for the correct orientation of the αC helix and the consequent stabilization of the salt bridge (Hohmann et al., 2017; Bojar et al., 2014; Huse and Kuriyan, 2002), this structural deficiency may be the primary cause of instability in this specific heterodimer, which may in fact be catalytically compromised.

We also critically evaluated the catalytic potential of the SbPSTOL1 heterotrimer, analyzing how its higher-order organization influences the accessibility of the active site. Stable αC-Glu ↔ β3-Lys salt bridges were formed in each SbPSTOL1 protein within the trimeric model throughout the simulation time. However, visual analysis of the trimeric assembly reveals a possibly significant steric restriction. The catalytic pockets of Sb03g031690 and Sb07g002840 are oriented in a “face-to-face” configuration, mutually obstructing each other. Consequently, within this specific trimeric arrangement, only the catalytic pocket of the Sb03g006765 subunit remains fully accessible and free from steric hindrance, suggesting that although the complex appears to be structurally stable, its catalytic competence may be functionally asymmetric.

The predicted structural contrast between OsPSTOL1 and the SbPSTOL1 proteins also raises evolutionary and functional questions regarding these kinases. OsPSTOL1 is predicted to be a cytosolic kinase with a stable, oligomerization-independent salt bridge, whereas the SbPSTOL1 proteins adopt a canonical receptor-like kinase architecture with a transmembrane helix and extracellular GUB_WAK and WAK-association domains that were conserved across the Andropogoneae. Because WAK-type ectodomains can bind cell-wall pectins (Kohorn and Kohorn, 2012), it is logical to hypothesize that the SbPSTOL1 proteins may couple the perception of apoplastic or cell-wall cues to intracellular signaling via ligand- or contact-induced oligomerization, whereas OsPSTOL1, in lacking these ectodomains, may act in a more constitutive and cell-autonomous manner. We speculate that this divergence could reflect distinct signaling strategies. For SbPSTOL1 proteins, an oligomerization-tunable membrane receptor may afford additional regulatory control coupling low P signaling and changes in root growth, enhancing P acquisition in low P soils (Hufnagel et al., 2014). In turn, the cytosolic configuration of rice OsPSTOL1 (Gamuyao et al., 2012) may represent an alternative, possibly derived state of the PSTOL1 kinases, whereby changes in root morphology may be totally or partially independent of low P-based signaling taking place in the cell surface. Divergence of OsPSTOL1 is supported by our phylogenetic analysis within the Andropogoneae, where OsPSTOL1 did not cluster with any of the PSTOL1-like proteins in the phylogeny. The corollary to this model is that, rather than the catalytic machinery itself, it is the surrounding architecture that may be responsible for PSTOL1 specialization, in line with the known repurposing of a conserved kinase domain under distinct regulatory logic imposed by its structural context (Taylor and Kornev, 2011). These interpretations remain hypotheses generated from *in silico* models and would require experimental and phylogenetic testing.

Finally, the activation model proposed here is entirely computational and remains hypothetical until experimentally validated. The predicted SbPSTOL1 interactions and their oligomerization-dependent activation could be tested by protein–protein interaction assays such as co-immunoprecipitation, bimolecular fluorescence complementation (BiFC) or split-luciferase complementation assays, the latter already applied to the related ZmWAKL–ZmWIK pair (Zhong et al., 2024). Site-directed mutagenesis of the predicted interface residues, particularly the αC-helix and activation-loop contacts, combined with *in vitro* kinase-activity assays comparing monomeric and oligomeric states, would test whether oligomerization is required to establish the αC-Glu ↔ β3-Lys salt bridge and enable catalytic competence. Although beyond the scope of the present computational study, these experiments outline a clear roadmap for validating our predictions.

## 5 Conclusion

This study establishes the first integrative structural framework for SbPSTOL1 proteins in *Sorghum bicolor*. SbPSTOL1 proteins are markedly different than OsPSTOL1 at the structural level suggesting a more autonomous function of OsPSTOL1, whereas SbPSTOL1 proteins appear to rely on signal perception and dimerization to perform their role in root morphology and architecture.

By systematically combining multimodal AI-based structural prediction with membrane-aware molecular dynamics simulations, oligomeric states of SbPSTOL1 proteins are more likely functional compared to monomeric states.

Importantly, our *in silico* results offer model-based hypotheses for RLK activation, suggesting that oligomer formation is associated with a conformational transition that exposes the catalytic cleft and enables the formation of the conserved αC-Glu ↔ β3-Lys salt bridge, a hallmark of kinase activation.

Beyond the specific case of SbPSTOL1, this work introduces a potentially transferable computational framework for modeling membrane-bound proteins. This framework integrates AI-driven modeling, interface-level analysis, and molecular dynamics simulations, with the final goal of investigating the challenging structures of membrane receptors.

## Data Availability

The authors declare that the data supporting the findings of this study are available within the paper and its **Supplementary Information files**. The amino acid sequences of three SbPSTOL1 homologs, Sb03g006765, Sb03g031690 and Sb07g002840 were retrieved from v1.4 of the sorghum genome available in the Phytozome database (https://phytozome-next.jgi.doe.gov/). In the most recent genome version, v3.1.1 and v5.1, the corresponding gene identifiers are Sobic.003G080700, Sobic.003G263400 and Sobic.007G032900. The OsPSTOL1 genomic sequence is available in GenBank under accession numbers BAK26566. The genomic sequences of ZmWAKL and ZmWIK used for analysis are available at GenBank, OQ421108 and OQ421112, respectively.
