# Supplementary material for: Artificial intelligence-based structural modeling of PSTOL1 receptor kinases in rice and sorghum
Source: Front Plant Sci. 2026 Sep 7;17:1850847. doi: 10.3389/fpls.2026.1850847 (PMC13590690; doi:10.3389/fpls.2026.1850847)
Supplement: Supplementary file 1 [file DataSheet1.docx]

**Supplementary Figures**

**Supplementary Figure S1.** Overview of the integrative computational pipeline used to characterize PSTOL1 receptor-like kinases (RLKs). The workflow proceeds through seven stages: (1) sequence analysis and functional annotation; (2) AI-based structure prediction of monomers and oligomers (AlphaFold2, AlphaFold3, RoseTTAFold v1/v2 and RoseTTAFold-All-Atom), evaluated with the pLDDT, pTM and ipTM confidence scores; (3) topology evaluation, retaining only models with spatially segregated extracellular, transmembrane and intracellular regions, using the experimentally characterized ZmWAKL–ZmWIK complex (Zhong et al., 2024); (4) membrane embedding in a POPC bilayer (CHARMM-GUI Membrane Builder, PPM 2.0/OPM); (5) heterodimer interface analysis (COCaDA contacts and the αC-Glu↔β3-Lys distance) with PyMOL curation; (6) region-resolved molecular dynamics simulations (NAMD3); and (7) integration of the topological, interface and dynamic evidence into a proposed, predicted mechanistic model. Stages 3–6 constitute the integrative modelling validation layer. All outputs are computational predictions.

**Supplementary Figure S2.** Maximum-likelihood phylogeny of the PSTOL1/LRK10 kinase domain in Andropogoneae proteins. PSTOL1 orthologs of the three SbPSTOL1 proteins were retrieved from Phytozome (Goodstein et al., 2012) for *Saccharum* (sugarcane, genome R570; Healey et al., 2024), *Andropogon gerardi* and *Miscanthus sinensis* (*Miscanthus* genome: Mitros et al., 2020) by reciprocal-best-hit BLASTp (Altschul et al., 1990). The phylogeny also includes the three sorghum SbPSTOL1 (McCormick et al., 2018) and rice OsPSTOL1 (Gamuyao et al., 2012) proteins. Additional LRK10/WAK-type kinases from maize (ZmWaklRLK1, GRMZM2G172396) and rice (LOC_Os01g49614), and two dicot sequences from tomato (Solyc02g086210) and *Arabidopsis* (AT5G39030), were included as outgroups; the tree was rooted using the two dicot (tomato and *Arabidopsis*) sequences. Only the kinase domain was used. Amino acid sequences were aligned against a central reference sequence (BLOSUM62) and a maximum-likelihood tree was inferred with VeryFastTree (Piñeiro et al., 2020) under the LG substitution model (Le and Gascuel, 2008) with 100 bootstrap resampling steps (Felsenstein, 1985). The SbPSTOL1 proteins clustered with other Andropogoneae proteins whereas OsPSTOL1 remained distinct. As an independent check, a Neighbor-Joining tree (Saitou and Nei, 1987) was also computed, and FastTree2 (Price et al., 2010), the algorithm underlying VeryFastTree, recovered the same grouping. In the tree, branch lengths (horizontal axis) are expressed as amino acid substitutions per site (evolutionary distance); numbers at the internal nodes are bootstrap support values (%), with only values ≥ 50 shown; the vertical axis only orders the protein entries and has no scale. Sequence labels are colored by genus.

**Supplementary Figure S3. Domain architecture of the *Andropogoneae* PSTOL1-like proteins, obtained from Phytozome as in Figure S2, with domains and sites predicted by InterProScan (Blum et al., 2021). Most orthologs share the extracellular GUB_WAK (IPR025287) and WAK-associated (IPR032872) domains and the intracellular protein-kinase domain (IPR000719/IPR011009) found in the SbPSTOL1 proteins; the transmembrane helix is also indicated (as predicted with Phobius (Käll et al., 2004)). Notably, all proteins carry the same conserved catalytic signatures, annotated identically by InterProScan: the ATP-binding site (IPR017441) and the Ser/Thr active site (IPR008271) and all are non-RD (HFD-type) kinases. The extracellular domains are therefore largely conserved in the *Andropogoneae*, except for the *Miscanthus sinensis* (05G208300 and 08G007500) and *Saccharum* R570 (08Cg037800) proteins, which lack or replace these extracellular domains by LysM, whereas OsPSTOL1 is the cytosolic exception.**

**Supplementary Figure S4. Molecular dynamics and distance-compatible salt bridges in Sb03g031690 × Sb07g002840.** (A) Global stability by region measured by Root Mean Square Deviation (RMSD) trajectories over a 120 ns simulation for the heterodimer, transmembrane domain (TM) intracellular domain (kinase) and extracellular domain. (B) Root Mean Square Fluctuation (RMSF) trajectories, with individual protein domains identified by horizontal bars above each graph. (C) Predicted integrity of the conserved salt bridge monitored by the distance between αC-Glu ↔ β3-Lys over time. The interaction remains consistently below the threshold of 4.0 Å (dashed line). SP: signal peptide.

**Supplementary Figure S5. Molecular dynamics and distance-compatible salt bridges in Sb03g006765 × Sb07g002840.** (A) Global stability by region measured by Root Mean Square Deviation (RMSD) trajectories over a 100 ns simulation for the heterodimer, transmembrane domain (TM), intracellular domain (kinase) and extracellular domain. (B) Root Mean Square Fluctuation (RMSF) trajectories, with individual protein domains identified by horizontal bars above each graph. (C) Protein integrity of the conserved salt bridge monitored by the distance between αC-Glu ↔ β3-Lys over time. Note the higher fluctuation in this complex compared to other heterodimers. SP: signal peptide.

**Supplementary Figure S6. Molecular dynamics and distance-compatible salt bridges in the SbPSTOL1 heterotrimer.** (A) Global stability by region measured by Root Mean Square Deviation (RMSD) trajectories over a 60 ns simulation for the heterotrimer, transmembrane domain (TM), intracellular domain (kinase) and extracellular domain. (B) Root Mean Square Fluctuation (RMSF) trajectories for the three partners (Sb03g006765, Sb03g031690 and Sb07g002840), with individual protein domains identified by horizontal bars above each graph. (C) Preditected integrity of the conserved salt bridge monitored by the distance between αC-Glu ↔ β3-Lys for all three subunits simultaneously. SP: signal peptide.

**Supplementary Videos**

**Supplementary Videos S1 - S4.** Animations of SbPSTOL1 and OsPSTOL1 monomers modeled with RoseTTAFold1 and embedded in a POPC-type plasma membrane using CHARM-Gui. The image is rotated to facilitate visualization of all sides of the protein. The entire protein is shown in gray, while the ATP-binding site, active site, and activation loop, which represent what we call the catalytic pocket, are shown in magenta, red, and cyan, respectively

**Supplementary Videos S5 - S8.**  Animations of SbPSTOL1 oligomers generated with Alphafold3 and inserted into a POPC-type membrane using CHARMM-GUI. The image has been rotated to facilitate visualization of all sides of the oligomer. In the model, we highlight the catalytic sites, ATP binding site, active site and activation loop in magenta, red, and cyan, respectively. The domains are also represented in the video, following this color pattern: GUB_WAK_Bind (Yellow), WAK_Assoc (Orange), Lysm_RLK (Green), Transmembra (Dark Gray), and Kinase (Blue).

**Supplementary Video S9**. Molecular dynamics of the Sb03g006765 x Sb03g031690 heterodimer inserted into the plasma membrane during a 300 ns trajectory. This animation shows the last 60 seconds of the total trajectory. The protein in blue is Sb03g006765 and the one in red is Sb03g031690. Available at: https://doi.org/10.6084/m9.figshare.31943460.

**Suplementary Tables**

**Supplementary Table S1.** Confidence scores of the predicted ZmWAKL and ZmWIK monomers with different structure prediction tools. pLDDT is reported on a 0 – 100 scale and pTM on a 0 – 1 scale; the metric shown are the same shown in Table 1. The predicted topological consistency of each model is described in the main text.

| **Software** | **Metrics** | **ZmWAKL** | **ZmWIK** |
| --- | --- | --- | --- |
| AlphaFold3 | pTM | 0.48 | 0.62 |
| AlphaFold2 | pLDDT | 75 | 83 |
| RoseTTAFold2 | pLDDT | 65 | 69 |
| RoseTTAFold-All-Atom | pLDDT | 65 | 71 |
| RoseTTAFold1 | pTM | 0.64 | 0.77 |

**Supplementary Table S2**. **Summary of intermolecular contacts in the modeled SbPSTOL1 complexes, calculated using the COCADA tool.**

| **Complex / Interface** | **Attractive Contacts (AT)** | **Salt Bridges (SB)** | **Repulsive Contacts (RE)** | **Hydrophobic Interactions (HY)** | **Hydrogen Bonds (HB)** | **Total Contacts** |
| --- | --- | --- | --- | --- | --- | --- |
| **Heterodimers** | | | | | | |
| Sb03g006765_Sb03g031690 | 6 | 4 | 5 | 58 | 34 | 107 |
| Sb03g006765_Sb07g002840 | 5 | 4 | 3 | 85 | 58 | 155 |
| Sb03g031690_Sb07g002840 | 8 | 3 | 8 | 97 | 42 | 158 |
| **Heterotrimer** | | | | | | |
| Sb03g006765–Sb03g031690 | 1 | 1 | 0 | 18 | 4 | 24 |
| Sb03g006765–Sb07g002840 | 7 | 3 | 3 | 70 | 39 | 122 |
| Sb03g031690–Sb07g002840 | 1 | 1 | 2 | 38 | 6 | 48 |

## **References**

Altschul, S. F., Gish, W., Miller, W., Myers, E. W., and Lipman, D. J. (1990). Basic local alignment search tool. J. Mol. Biol. 215, 403–410. doi: 10.1016/S0022-2836(05)80360-2

Felsenstein, J. (1985). Confidence limits on phylogenies: an approach using the bootstrap. Evolution 39, 783–791. doi: 10.1111/j.1558-5646.1985.tb00420.x

Goodstein, D. M., Shu, S., Howson, R., Neupane, R., Hayes, R. D., Fazo, J., et al. (2012). Phytozome: a comparative platform for green plant genomics. Nucleic Acids Res. 40, D1178–D1186. doi: 10.1093/nar/gkr944

Healey, A. L., Garsmeur, O., Lovell, J. T., Sreedasyam, A., Jenkins, J., Manners, J. M., et al. (2024). The complex polyploid genome architecture of sugarcane. Nature 628, 804–810. doi: 10.1038/s41586-024-07231-4

Käll, L., Krogh, A., and Sonnhammer, E. L. L. (2004). A combined transmembrane topology and signal peptide prediction method. *J. Mol. Biol.* 338, 1027–1036. doi: 10.1016/j.jmb.2004.03.016

Le, S. Q., and Gascuel, O. (2008). An improved general amino acid replacement matrix. Mol. Biol. Evol. 25, 1307–1320. doi: 10.1093/molbev/msn067

Mitros, T., Session, A. M., James, B. T., Wu, G. A., Belaffif, M. B., Clark, L. V., et al. (2020). Genome biology of the paleotetraploid perennial biomass crop Miscanthus. Nat. Commun. 11:5442. doi: 10.1038/s41467-020-18923-6

Piñeiro, C., Abuín, J. M., and Pichel, J. C. (2020). Very Fast Tree: speeding up the estimation of phylogenies for large alignments through parallelization and vectorization strategies. Bioinformatics 36, 4658–4659. doi: 10.1093/bioinformatics/btaa582

Price, M. N., Dehal, P. S., and Arkin, A. P. (2010). FastTree 2 — approximately maximum-likelihood trees for large alignments. PLoS ONE 5:e9490. doi: 10.1371/journal.pone.0009490

Saitou, N., and Nei, M. (1987). The neighbor-joining method: a new method for reconstructing phylogenetic trees. Mol. Biol. Evol. 4, 406–425. doi: 10.1093/oxfordjournals.molbev.a040454
